# Multi-omics analysis of long COVID (post-COVID-19 condition) reveals persistent mitochondrial dysfunction, suppressed oxidative phosphorylation, and immune dysregulation

**DOI:** 10.3389/fimmu.2026.1776555

**Published:** 2026-05-21

**Authors:** Alexia Tasoula, Shehbeel Arif, Ethan Waisberg, Lucas Bauer, Elizabeth Aslinger, Joseph W. Guarnieri

**Affiliations:** 1 Guarnieri Research Group LLC, Philadelphia, PA, United States; 2 Ohio University, Heritage College of Osteopathic Medicine, Athens, OH, United States; 3 Drexel University College of Medicine, Philadelphia, PA, United States; 4 University of Cambridge, Department of Medical Genetics, Cambridge, United Kingdom; 5 North Carolina State University, Department of Molecular and Structural Biochemistry, Raleigh, NC, United States; 6 Aslinger Scientific Consulting, Round Rock, TX, United States; 7 Blue Marble Space Institute of Science, Seattle, WA, United States

**Keywords:** bioenergetic dysfunction, SARS-CoV-2 infection, post-acute sequelae of COVID-19 (PASC), mitochondrial stress response, systemic inflammation, transcriptomic reprogramming, metabolic remodeling

## Abstract

**Introduction:**

Post-COVID Syndrome (PCS), or long-COVID, is a major public health burden, but its underlying mechanisms remain poorly understood. Because acute SARS-CoV-2 infection induces marked suppression of mitochondrial oxidative phosphorylation (OXPHOS), we investigated whether persistent immunometabolic remodeling is a recurring transcriptional, metabolic, and proteomic feature of PCS.

**Methods:**

We performed an integrated multi-omics analysis of transcriptomic, proteomic, and metabolomic datasets across multiple tissues from Syrian hamster models and human cohorts spanning acute infection through post-acute and PCS stages extending up to 12 months post-infection.

**Results:**

Across species and tissues, we observed overlapping signatures of mitochondrial dysfunction, including sustained suppression of OXPHOS, activation of mitochondrial stress responses, and enrichment of inflammatory pathways. Skeletal muscle exhibited the most pronounced and persistent mitochondrial repression in both hamsters and PCS patient biopsies, consistent with fatigue-associated phenotypes. Hamster heart and kidney tissues also showed persistent OXPHOS suppression, while lung tissue demonstrated prolonged inflammatory signaling despite partial metabolic recovery. In the nervous system, transcriptional profiles revealed region-specific patterns, including persistent cortical mitochondrial repression and partial recovery in sensory-associated regions. Peripheral blood mononuclear cells (PBMCs) transcriptomics and serum metabolic datasets suggested prolonged downregulation of OXPHOS-associated programs up to 12 months post-infection, potentially contributing to persistent immune dysregulation in susceptible individuals with underlying conditions. Longitudinal serum proteomics in PCS patients revealed sustained mitochondrial stress responses, increased oxidative stress signatures, and persistent immune activation at 1 and 6 months post-infection compared to recovered controls.

**Discussion:**

Together, these multi-omics results identify persistent mitochondrial repression and immune dysregulation as recurring features across PCS-associated datasets, providing a framework linking bioenergetic dysfunction with chronic immune activation and supporting future mechanistic and therapeutic investigation.

## Highlights

- Persistent mitochondrial and immune alterations are observed across multiple tissues in PCS-associated datasets.
- Serum proteomic profiles suggest ongoing mitochondrial stress and immune activation in PCS.
- PCS skeletal muscle shows sustained OXPHOS suppression, consistent with fatigue-associated phenotypes.
- Brain regions exhibit heterogeneous metabolic recovery, with persistent cortical mitochondrial repression observed in PCS models.

## Introduction

Long-COVID, also known as Post-COVID Syndrome (PCS), is a chronic, multisystem condition that arises in select individuals following acute SARS-CoV-2 infection. Hallmark symptoms, including memory impairment, concentration difficulties, chronic fatigue, muscle weakness, and cardiovascular complications such as arrhythmias and myocarditis, can persist for months to years after viral clearance (1–5). Epidemiological studies estimate that 10–26% of infected individuals develop PCS, translating to a global burden of up to 400 million people to date. The economic impact is similarly vast, exceeding $1 trillion USD per year worldwide, roughly 1% of global GDP (1, 6, 7). Despite its increasing prevalence, there are currently no FDA-approved treatments or validated biomarkers for PCS, underscoring a critical gap in our understanding and ability to intervene (1, 6, 7). Despite extensive investigation, the molecular mechanisms linking acute SARS-CoV-2 infection to persistent multi-system dysfunction remain poorly defined. In particular, it remains unclear whether a unifying biological process underlies the diverse clinical manifestations observed across patient populations.

Clinically, PCS shares similarities with syndromes associated with mitochondrial dysfunction, including Myalgic Encephalomyelitis/Chronic Fatigue Syndrome (ME/CFS), fibromyalgia, and postural orthostatic tachycardia syndrome (POTS) (1–5). Shared features include persistent low-grade inflammation and prolonged immune dysregulation that extend well beyond clinical recovery, particularly in high-energy-demand tissues such as the brain (8–10), heart (1, 3, 4, 11, 12), skeletal muscle (13–15), and immune system (16–19). This dysregulation is characterized by sustained immune activation (1, 3, 4, 8–20), accumulation of senescence markers (21, 22), and sustained cytokine elevation in plasma and serum (9, 23, 24). Previously, our group and others have shown that SARS-CoV-2 profoundly disrupts mitochondrial function throughout infection, with these effects extending well beyond viral clearance (3, 25–30).

Given these clinical parallels and emerging mechanistic evidence, mitochondrial alterations have been associated with metabolic and immune dysregulation observed in PCS (3, 6, 31). Mitochondria generate ~90% of cellular ATP through OXPHOS, a tightly regulated process that requires coordinated expression between nuclear (nDNA) and mitochondrial (mtDNA) genomes. Tissues with high energy demands, such as the brain, heart, skeletal muscle, and immune cells, are particularly sensitive to mitochondrial stress. Impaired OXPHOS leads to ATP depletion and the generation of mitochondrial reactive oxygen species (mROS). Elevated mROS stabilizes hypoxia-inducible factor 1 alpha (HIF-1α), promoting a metabolic shift toward glycolysis, a short-term adaptation that ultimately fails under chronic stress (3, 32, 33).

Elevated mROS also triggers protective responses, including antioxidant defenses, mitophagy to remove damaged mitochondria, and stress signaling. However, under persistent stress, these systems become overwhelmed, resulting in the damage and release of mitochondrial damage-associated molecular patterns (mtDAMPs), including mtDNA, mitochondrial double-stranded RNA (mtdsRNA), and lipids such as cardiolipin. Due to their bacterial origin, when exposed to the cytosol, these molecules are potent immunostimulants, engaging pattern recognition receptors (PRRs), driving interferon and cytokine responses, and perpetuating systemic inflammation (3, 32–36).

SARS-CoV-2 infection has been shown to impair host mitochondria function from acute to post-acute phases (3, 25–30, 37). During acute illness, host bioenergetics are rapidly reprogrammed. SARS-CoV-2 impairs mitochondrial OXPHOS via multiple mechanisms, including direct viral interference with mitochondrial machinery (3, 25, 37), miR-2392 expression (26, 38), transcriptional repression of mitochondrial genes (3, 26–28), and activation of host pathways (e.g., HIF-1α, mTORC1) that favor aerobic glycolysis (3, 26, 27, 39, 40). This shift elevates mROS, reinforcing a pro-glycolytic, “Warburg-like” metabolic state that supports the biosynthetic demands of infected cells and enhances viral replication (3, 27). In parallel, elevated mROS trigger the release of mtDAMPs, amplifying systemic inflammation and contributing to cytokine storm severity (3, 25, 27). Notably, mitochondria-targeted antioxidants reduce replication and disease severity, highlighting mitochondrial stress as a key factor in SARS-CoV-2 virulence (27, 41). Collectively, these findings support the hypothesis that prolonged mitochondrial dysfunction may contributor to systemic pathology rather than a transient consequence of infection.

PCS are a clinically defined subset of post-infectious outcomes, marked by persistent or relapsing symptoms that endure for at least three months after acute SARS-CoV-2 infection. In this study, we explicitly differentiate PCS from general post-infectious symptom persistence (1–5). To characterize the metabolic and immune changes in PCS models, we conducted a cross-species, multi-tissue study integrating transcriptomic, proteomic, and metabolomic analyses from human and Syrian hamster samples collected during acute, post-acute, and PCS phases (14–16, 20, 22, 42–44). Hamster analyses spanned skeletal muscle, heart, kidney, lung, liver, and eight brain regions at 3-, 31-, and 61-days post-infection (dpi) (15, 20). Human datasets included peripheral blood mononuclear cells (PBMCs) from individuals following SARS-CoV-2 infection (16), along with PCS skeletal muscle (14) and plasma samples (43, 44) collected between 1 and 24 months post-infection.

By integrating these heterogeneous datasets within a unified analytical framework and directly comparing individuals who recovered with those experiencing persistent or more severe symptoms, we identify recurrent immunometabolic signatures across tissues and multiple omic modalities. This approach extends prior single-cohort studies by highlighting shared patterns across independent datasets. Collectively, these findings are consistent with a model in which incomplete restoration of mitochondrial bioenergetic pathways is associated with sustained immune dysregulation in several organs, offering a potential framework for understanding the systemic features of PCS.

## Results

### Tissue-specific mitochondrial suppression and immune dysregulation persist across multiple organs following SARS-CoV-2 infection

#### Tissue-specific metabolic and immune responses in heart, kidney, and lung tissues after SARS-CoV-2 infection

**Figure 1:**
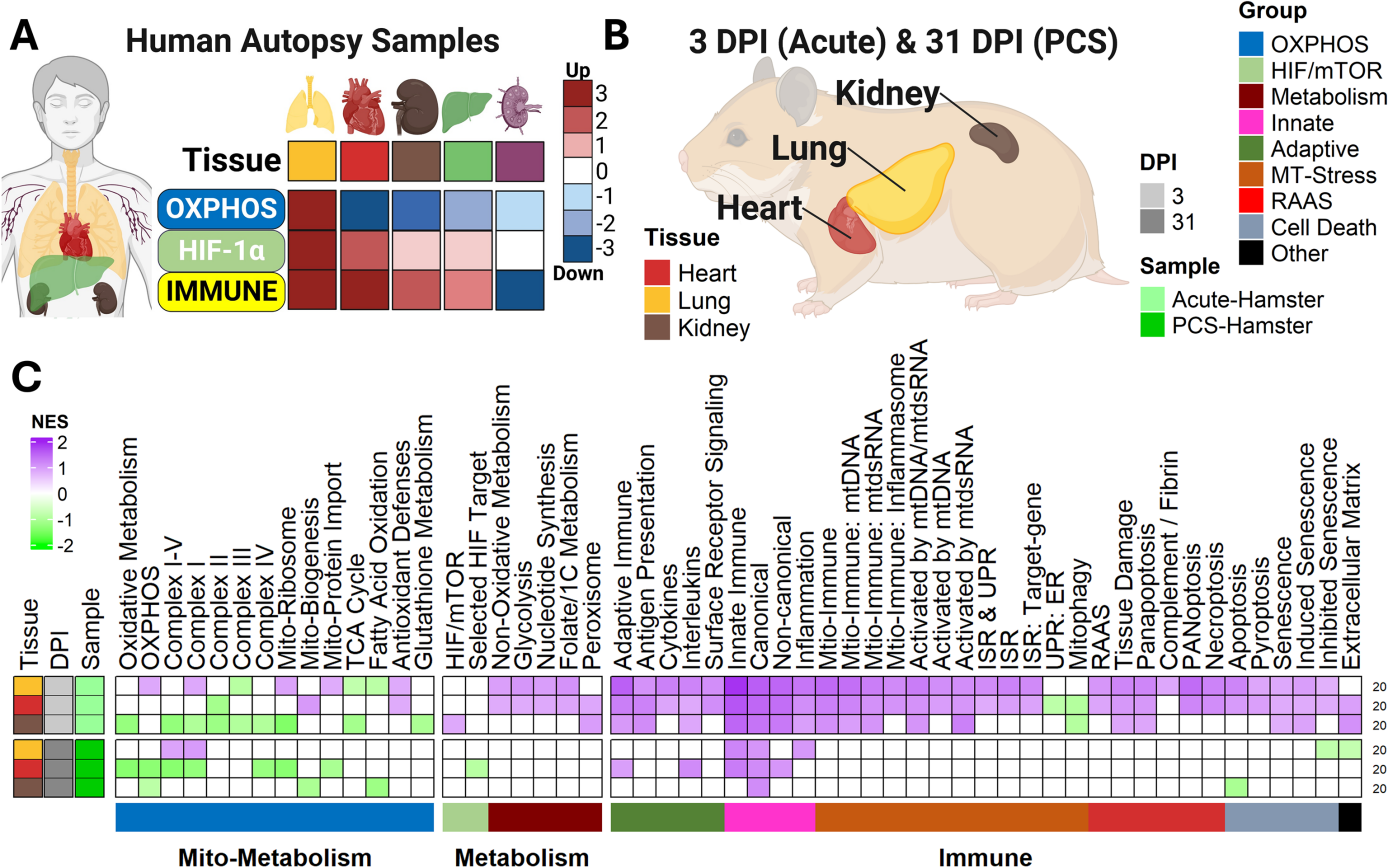
Tissue-wide profiling shows cardiac mitochondrial dysfunction and pulmonary immune activation in a PCS hamster model. **(A)** Summary of transcriptomic findings from COVID-19 autopsy samples (26, 28). **(B)** Overview of hamster tissue RNA-seq datasets analyzed. **(C)** Heatmap of normalized enrichment scores (NES)> from Gene Set Enrichment Analysis (GSEA) using custom pathways, showing transcriptomic alterations. False Discovery Rate (FDR) threshold = 0.25. RNA-seq sample sizes for heart, kidney and lung tissues were as follows: Acute-phase (3 dpi): SARS-CoV-2-infected (n=3), Mock (n = 4). Post-acute phase (31 dpi): SARS-CoV-2-infected (n=3), Mock (n = 3). GSEA comparisons: SARS-CoV-2–infected vs. Mock. All n values represent biologically independent animals.

In our previous work, we demonstrated that COVID-19 autopsy tissues—including the heart, kidney, liver, and lymph nodes—exhibited prolonged inhibition of OXPHOS transcripts (26, 28) (**Figure 1A**). Notably, these transcriptional changes persisted despite the absence of detectable viral transcripts, suggesting that SARS-CoV-2 infection is associated with sustained mitochondrial and immune dysregulation even after viral clearance. This repression was accompanied by increased expression of immune and HIF-1α transcripts across all tissues except the lymph nodes. In the lymph nodes, there was a significant decrease in immune gene transcription and a marked upregulation of the renin-angiotensin-aldosterone system (RAAS) pathway, which coincided with histological scarring. Conversely, lung tissue showed recovery of OXPHOS transcripts but exhibited the highest level of immune activation, characterized by significantly elevated mitochondrial immune and stress responses (26, 28).

These findings suggest that persistent immunometabolic alterations can remain in viral patients long after viral clearance, suggesting that sustained activation of these pathways may contribute to PCS. However, because these samples were derived from individuals who succumbed to acute COVID-19, the observed mitochondrial and immune alterations likely represent the upper bounds of SARS-CoV-2–induced tissue stress and inflammatory activation rather than PCS-specific pathology. Unlike severe human cases, PCS patients do not succumb to infection, limiting access to post-acute human tissues. To investigate these processes in a model more reflective of post-acute disease, we analyzed a hamster model of PCS.

The Syrian hamster is a well-established model that recapitulates key features of mild human PCS (45). These animals, being naturally susceptible to SARS-CoV-2, develop a non-lethal, self-limiting disease, reaching peak lung viral loads at the acute phase (3 dpi) and achieving complete viral clearance in all the tissues by 5 dpi (20, 26, 28). The post-acute/chronic phase (31 dpi) displays respiratory dysfunction and neurological impairment at 31 dpi (20). To build on these findings we performed pathway analysis on RNA-seq data from the heart, kidney, and lungs of SARS-CoV-2-infected hamsters (**Figures 1B, C****;** **Supplementary Figure S1**).

During the acute phase (3 dpi), we observed upregulation of mitochondrial ribosomal genes in the lungs and heart, along with signatures of enhanced mitochondrial biogenesis in the heart and kidneys (**Figure 1C**). Non-oxidative metabolic genes were also upregulated in the heart and lung, suggesting a shift away from oxidative metabolism that was previously shown to be crucial for facilitating viral replication in the lungs (3, 27, 46). Robust innate immune activation was also observed across all tissues, with enrichment of pathways related to mtdsRNA and mtDNA signaling. This response was most pronounced in the lungs, followed by the heart and the kidneys. RAAS and senescence-associated pathways were activated in the lungs, heart, and kidneys, while PANoptosis-related signatures were strongly induced in the lungs and heart (**Figure 1C**).

By the post-acute phase (31 dpi), lung metabolism normalized, aligning with prior human data showing mitochondrial recovery after severe COVID-19 (26) (**Figure 1C**). However, immune gene expression and mitochondrial stress responses in the lung remained elevated, indicating ongoing inflammatory signaling. In contrast, heart and kidney tissues continued to exhibit suppressed OXPHOS expression, reflecting sustained bioenergetic impairment. While immune signatures had resolved in the kidney, they remained modestly elevated in the heart. The sustained mitochondrial suppression signaling in the heart suggests a greater vulnerability to long-term dysfunction in cardiovascular tissues, and the persistent activation of mitochondrial stress responses in the lung indicates a distinct vulnerability to chronic immune dysregulation.

#### Persistent mitochondrial suppression in skeletal muscle links long-COVID to ME/CFS

**Figure 2:**
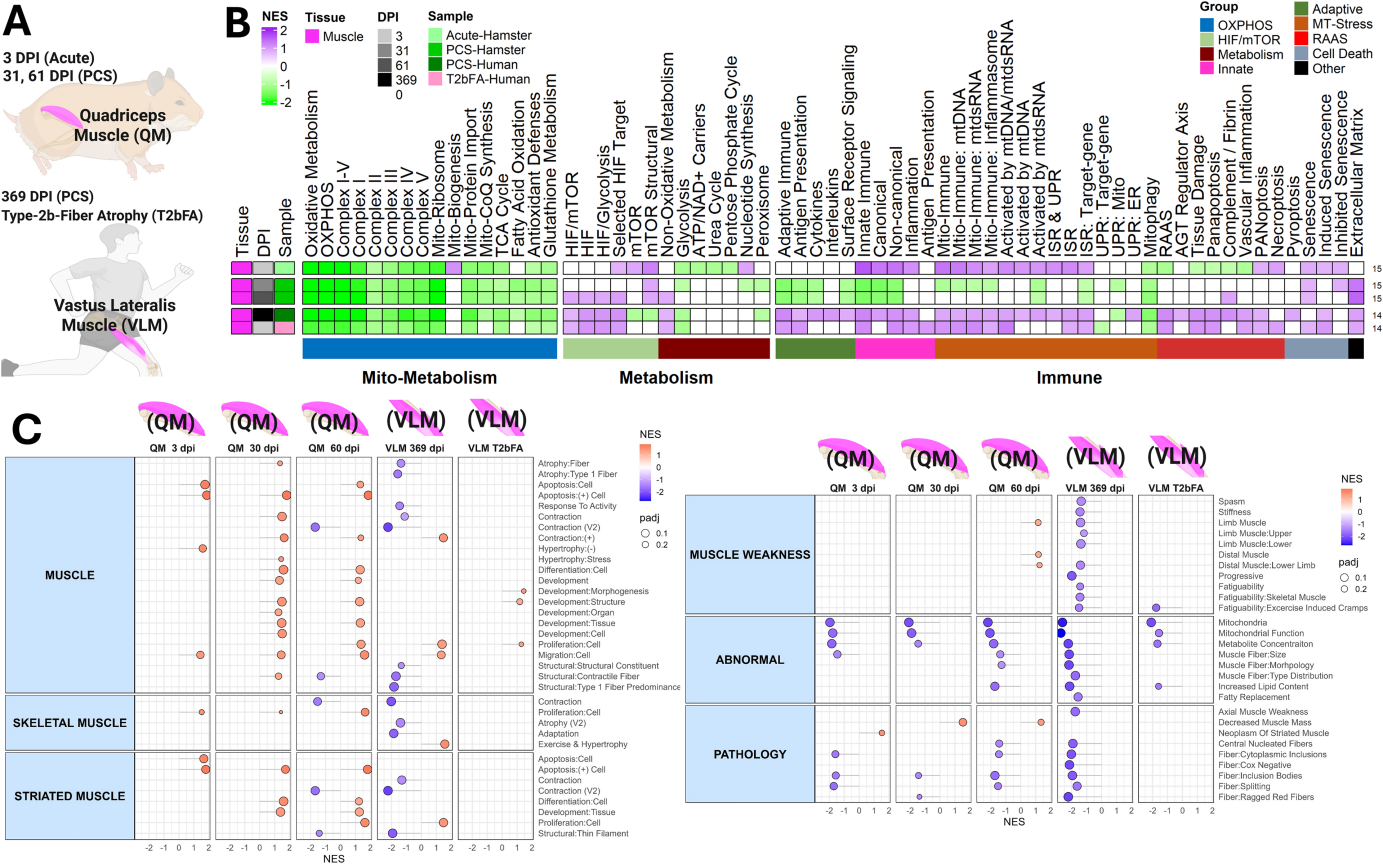
Persistent skeletal muscle mitochondrial impairment links post-COVID fatigue to chronic fatigue syndrome. **(A)** Overview of hamster and human muscle tissue RNA-seq datasets analyzed. **(B)** Heatmap and **(C)** lollipop plots of NES from GSEA using **(B)** custom pathways or **(C)** muscle function–related pathways, highlighting transcriptomic alterations. FDR threshold = 0.25. RNA-seq samples were as follows: Hamster skeletal muscle: SARS-CoV-2–infected at 3 dpi (n = 3), 31 dpi (n = 3), and 61 dpi (n = 3), Mock (n = 3). GSEA comparisons: SARS-CoV-2–Infected vs. Mock. Human skeletal muscle: PCS patients (n = 11), Type IIb fibers atrophy patients (T2bFA) (n = 8), Control Patients (n = 8). GSEA comparisons: PCS vs. Control and T2bFA vs. Control. All n values represent biologically independent samples.

To investigate chronic fatigue, we analyzed transcriptomic data from hamster quadriceps muscles collected at 3, 31, and 61 dpi (15). To assess translational relevance in muscle tissue to humans, we also examined vastus lateralis muscle biopsies from individuals with PCS that developed chronic fatigue syndrome (PCS-CFS) up to 1-year (369 dpi) post-infection, comparing them with Type 2B muscle fiber atrophy (T2bFA) patients that also suffer from CFS (T2bFA-CFS) without prior SARS-CoV-2 infection (14) (**Figure 2A**).

At 3 dpi, hamster skeletal muscle, displayed a downregulation of OXPHOS, and upregulation of innate immune and mitochondrial stress response transcripts (**Figure 2B****;** **Supplementary Figure S2**). OXPHOS gene expression remained suppressed at 31 and 61 dpi, with the later time points also showed persistent extracellular matrix remodeling and, by 61 dpi, increased expression of HIF and mTOR pathways.

In human skeletal muscle, both PCS-CFS and T2bFA-CFS patients exhibited similar transcriptional signatures, including downregulated OXPHOS and elevated HIF signaling. However, PCS-CFS samples also showed heightened activation of the integrated stress response (ISR), unfolded protein response (UPR), and both innate and adaptive immune signaling. T2bFA-CFS patients also exhibited predominant upregulation of mitochondrial stress, ISR, and induced senescence transcripts (**Figure 2B**). Notably, several immune and stress-response pathways were more pronounced in humans than in hamsters—differences likely reflecting variation in sampling intervals, disease severity, and species-specific immune adaptation.

To provide context for these findings, we developed a custom list of pathways from publicly available gene lists related to muscle function. We then organized these pathways into enriched terms within functional clusters and conducted GSEA analysis (**Figure 2C****;** **Supplementary Table S1**). In hamster skeletal muscle we observed a progressive disruption of muscle function-related pathways from 3 to 61 dpi. At 3 dpi, pathways related to apoptosis and proliferation were enriched. By 31 dpi, pathways associated with development and differentiation were upregulated, and muscle mass pathways were downregulated. These alterations were maintained at 61 dpi, which also displayed a suppression of contraction-related pathways along with an increase in pathways linked to abnormal pathology, consistent with a decline in muscle function.

In humans, consistent with their pathological phenotype, PCS-CFS samples showed significant downregulation of mitochondrial and contraction pathways, resembling the changes seen in hamster muscles at 61 dpi. T2bFA-CFS samples showed upregulation of developmental and proliferation pathways, alongside a downregulation of mitochondrial function pathways, consistent with the trends seen across all PCS muscle samples (**Figure 2C**). Notably, the overlap between PCS-CFS and T2bFA-CFS was more pronounced at the level of core transcriptional programs (**Figure 2B**) than at the pathway level (**Figure 2C**), indicating that shared mitochondrial repression may be accompanied by context-specific secondary remodeling programs. Overall, this research suggests that the persistent inhibition of OXPHOS transcripts is linked to progressive changes in pathways associated with muscle function decline and fatigue in PCS.

#### A neuroinflammatory signature and metabolic disruption underlie neurological symptoms

Neurological symptoms are among the most debilitating and persistent in PCS, encompassing anosmia, dysgeusia, brain fog, fatigue, and mood disturbances. These symptoms are thought to arise from a combination of direct viral effects, chronic neuroinflammation, and altered neuro-metabolism (9, 21, 22, 47–49). To investigate, we analyzed RNA-seq data from SARS-CoV-2–infected Syrian hamsters at acute (3 dpi) and chronic (31 dpi) time points across key sensory and cognitive brain regions (20).

**Figure 3:**
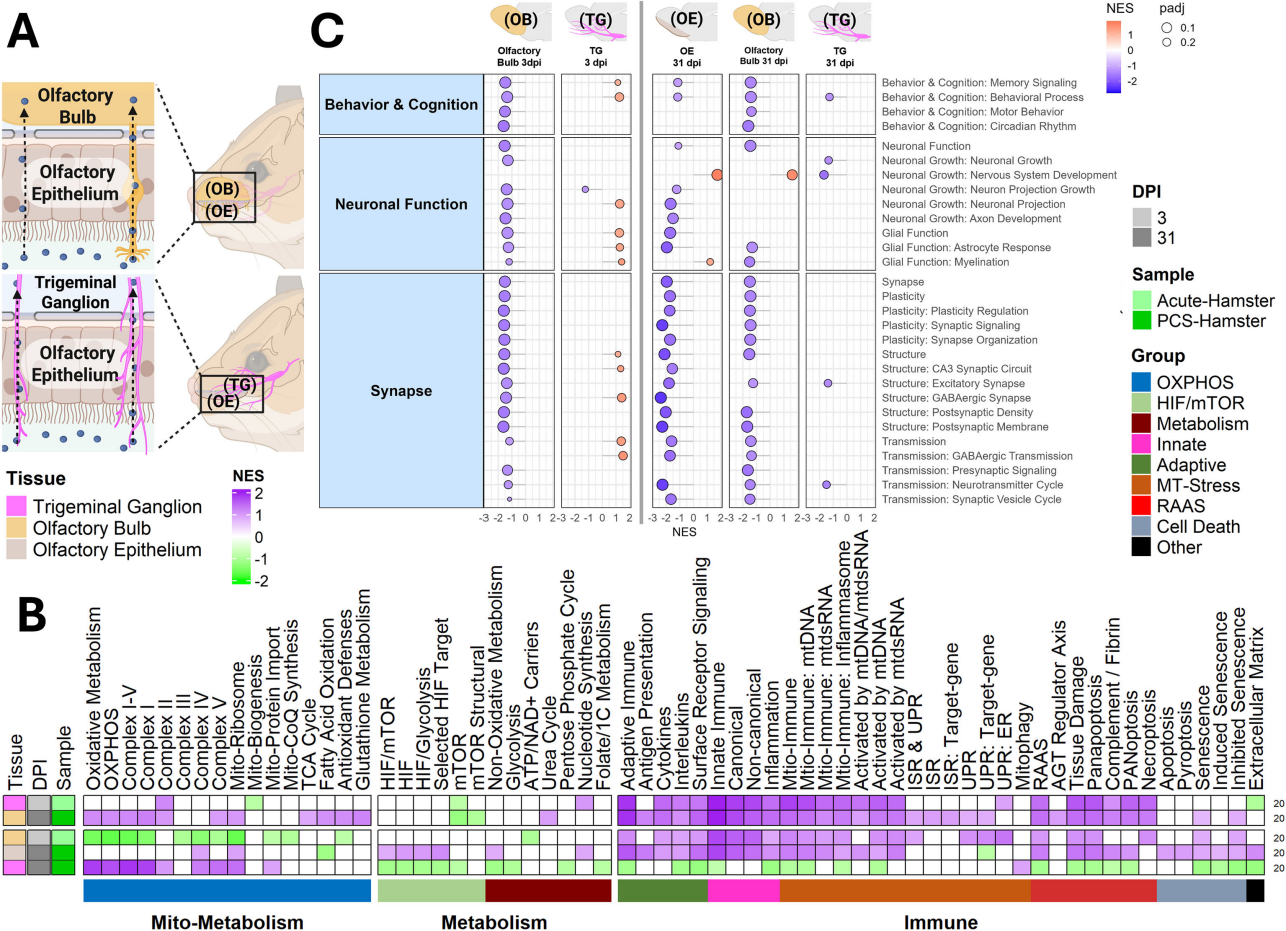
Olfactory circuit mitochondrial stress and immune activation drive sensory deficits in PCS. **(A)** Overview of hamster olfactory neural tissue RNA-seq datasets analyzed. **(B)** Heatmap and **(C)** lollipop plots of NES from GSEA using **(B)** custom pathways or **(C)** neuro function–related pathways, showing transcriptomic changes. FDR threshold = 0.25. RNA-seq samples were as follows: Olfactory Bulb: SARS-CoV-2-infected 3 dpi (n = 3), Mock 3 dpi (n = 3), SARS 31 dpi (n = 3), Mock 31 dpi (n = 3). Olfactory Epithelium: SARS 31 dpi (n = 4), Mock 31 dpi (n = 4). Trigeminal Ganglia: SARS 3 dpi (n = 3), Mock 3 dpi (n = 3), SARS 31 dpi (n = 5), Mock 31 dpi (n = 5). GSEA comparisons: SARS-CoV-2–Infected vs. Mock. All n values represent biologically independent animals.

To model sensory deficits, we profiled the olfactory epithelium (OE), olfactory bulb (OB), and trigeminal ganglia (TG), tissues responsible for smell and facial sensation (**Figure 3A**). The OE and OB are first-line sensory interfaces directly exposed to inhaled pathogens, with the OE acting as a mucosal immune sensor and the OB integrating signals via olfactory neurons. By contrast, the TG is more insulated but acts as a secondary sensory relay responsive to both peripheral and central signals (50).

During the acute phase (3 dpi), all sensory tissues displayed robust mitochondrial stress and innate immune activation (**Figure 3B**), consistent with systemic inflammatory signaling observed in peripheral tissues (**Figures 1**, **2**). In addition, the OB and TG showed strong induction of tissue damage pathways and PANoptosis-associated genes, alongside suppression of OXPHOS and peroxisomal metabolism. By 31 dpi, olfactory tissues remained inflamed, with the OE exhibiting persistent HIF activation and suppression of fatty acid oxidation (FAO). In contrast, immune signaling in the TG was largely resolved, while OXPHOS and mitophagy pathways were upregulated, indicating a regenerative mitochondrial response (**Figure 3B****;** **Supplementary Figure S3**).

To place these changes in a functional context, we again generated a custom list of pathways by combining publicly available gene lists related to brain function and performed GSEA analysis (**Figure 3C****;** **Supplementary Table S1**). This revealed sustained suppression of neuronal signaling and synaptic function in the OE and OB from 3 to 31 dpi, providing a mechanistic basis for persistent anosmia and sensory dysfunction in PCS. TG, in contrast, showed only mild, transient changes, aligning with its trajectory toward recovery. Collectively, these findings indicate that the olfactory system, particularly the OE and OB, undergoes early mitochondrial dysfunction and prolonged immune activation, processes that, if unresolved, may underlie long-term sensory deficits observed in post-acute phases.

**Figure 4:**
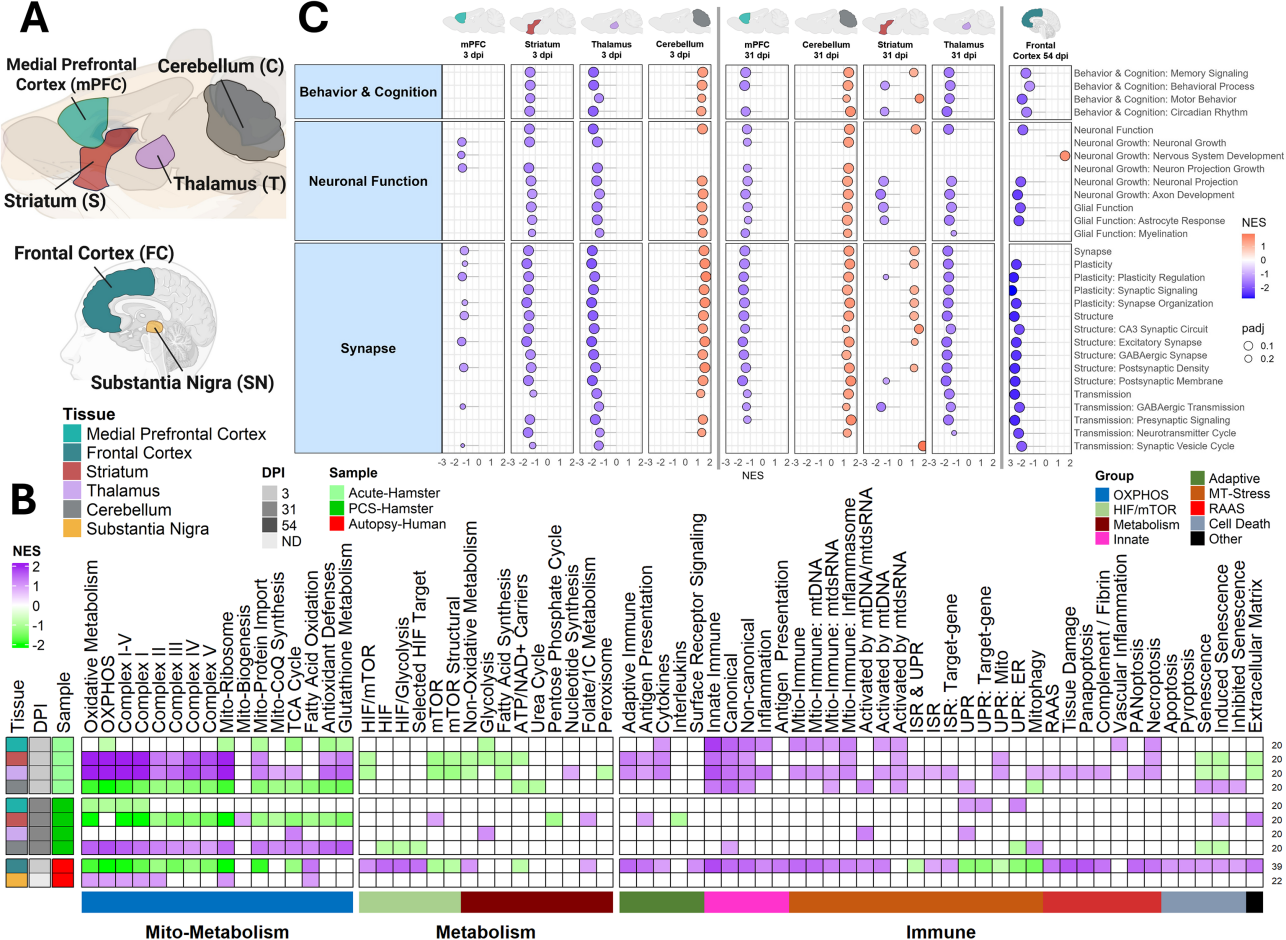
Frontal cortex neuroimmune and metabolic dysregulation highlights PCS vulnerability. **(A)** Overview of internal hamster and human neural tissue RNA-seq datasets analyzed. **(B)** Heatmap and **(C)** lollipop plots of NES from GSEA using **(B)** custom pathways or **(C)** neuro function–related pathways, showing transcriptomic alterations. FDR threshold = 0.25. RNA-seq sample sizes were as follows. Human brain tissues: Frontal cortex: COVID-19 autopsy (n = 20) and non-COVID autopsy controls (n = 22); substantia nigra: COVID-19 autopsy (n = 6) and non-COVID autopsy controls (n = 3). GSEA comparisons: COVID-19 autopsy vs. non-COVID autopsy controls. Hamster brain regions: 3 dpi groups included mPFC, striatum, thalamus, and cerebellum with SARS-CoV-2–infected (n = 3) and Mock (n = 3). 31 dpi groups included SARS-CoV-2–infected mPFC (n = 3), striatum (n = 4), thalamus (n = 3), and cerebellum (n = 5), with Mock (n = 5). GSEA comparisons: SARS-CoV-2–infected vs. Mock. All n values represent biologically independent animals.

To investigate cognitive and affective symptoms, we analyzed the striatum, thalamus, medial prefrontal cortex (mPFC), and cerebellum, regions involved in motor, executive, and emotional functions (20) (**Figure 4A**). Unlike the olfactory tissues, these brain regions are normally protected by the blood–brain barrier (BBB), making them less susceptible to immune activation unless the BBB integrity is compromised (9). Despite this protection, at 3 dpi, all brain regions showed a robust upregulation of innate immunity and mitochondrial stress pathways, raising the possibility of altered BBB integrity (**Figure 4B**). The thalamus also showed upregulation of PANoptosis and the cerebellum activated senescence pathways. The mPFC and cerebellum exhibited reduced OXPHOS, while the striatum and thalamus showed elevated OXPHOS transcripts. By 31 dpi, the striatum, thalamus, and cerebellum showed reversal of early responses, with decreased immune activity, consistent with greater BBB integrity. This reversal in immune response was associated with a reversal in metabolic response, with the striatum and thalamus now displaying reduced OXPHOS transcripts, and cerebellum upregulation. In contrast, the mPFC worsened, with stronger repression of OXPHOS transcripts (**Figure 4B**; **Supplementary Figure S4**).

Immune and metabolic changes were accompanied by altered neuronal function (**Figure 4C**). At 3 dpi, immune activation and mitochondrial stress correlated with reduced neuronal signaling in the thalamus, striatum, and mPFC. By 31 dpi, persistent OXPHOS inhibition in the mPFC aligned with suppressed neuronal pathways. The striatum and thalamus recovered both metabolically and functionally. Interestingly, the cerebellum showed enhanced neuronal pathways at 3 dpi but suppression at 31 dpi, suggesting incomplete functional recovery, despite greater metabolic recovery later (**Figure 4C**).

Collectively, our hamster model suggests that while the initial response to SARS-CoV-2 is a systemic, multi-organ inflammatory event, the long-term sequelae are defined by profound, tissue-specific bioenergetic deficits. The persistent mitochondrial suppression in high-energy tissues like skeletal muscle, heart, and specific brain regions may contribute to fatigue, exercise intolerance, and neurological symptoms of PCS. These findings in a controlled model raise the question of whether a similar, persistent pathogenic state can be identified in human patients experiencing PCS.

### Human multi-omics evidence suggests persistent mitochondrial suppression after SARS-CoV-2 infection

#### Human COVID-19 autopsy data support region-specific neuroimmune and bioenergetic disruption

Building on our previous observations of persistent OXPHOS suppression across multiple COVID-19 autopsy tissues (26, 28) (**Figure 1A**), we next examined whether similar mitochondrial and immune transcriptional programs are evident in the CNS. To do so, we analyzed publicly available RNA-seq datasets from human brain autopsy samples, specifically the striatum and frontal cortex, collected from patients who died from acute COVID-19 (21, 22, 42) (**Figure 4A**). While these severe cases may reflect intensive care units (ICU)-associated exposures (e.g., hypoxia, ventilation, sedation), the inclusion of non-infected autopsy controls provides a important baseline for interpreting COVID-19-associated transcriptional changes. In the frontal cortex, we observed pronounced inhibition of OXPHOS transcripts, increased HIF and mTOR signaling, and widespread immune activation, without clear induction of compensatory mitochondrial stress responses—findings consistent with inflammatory remodeling and possible BBB disruption (**Figure 4B****;** **Supplementary Figure S4**). In contrast, the substantia nigra displayed elevated OXPHOS-associated transcripts with minimal immune activation, suggesting regional metabolic resilience. Functionally, persistent OXPHOS inhibition in the frontal cortex coincided with suppression of neuronal and synaptic signaling pathways, whereas the substantia nigra exhibited few functional pathway changes (**Figure 4C**). Together, these results are consistent with region-specific metabolic and immune remodeling in the human brain during severe COVID-19, providing context for how acute neuroimmune stress may increase neurological vulnerability and potentially predispose susceptible individuals to persistent post-acute symptoms.

#### Mitochondrial transcriptional inhibition persists for months in PBMCs after infection in severe COVID-19 patients

**Figure 5:**
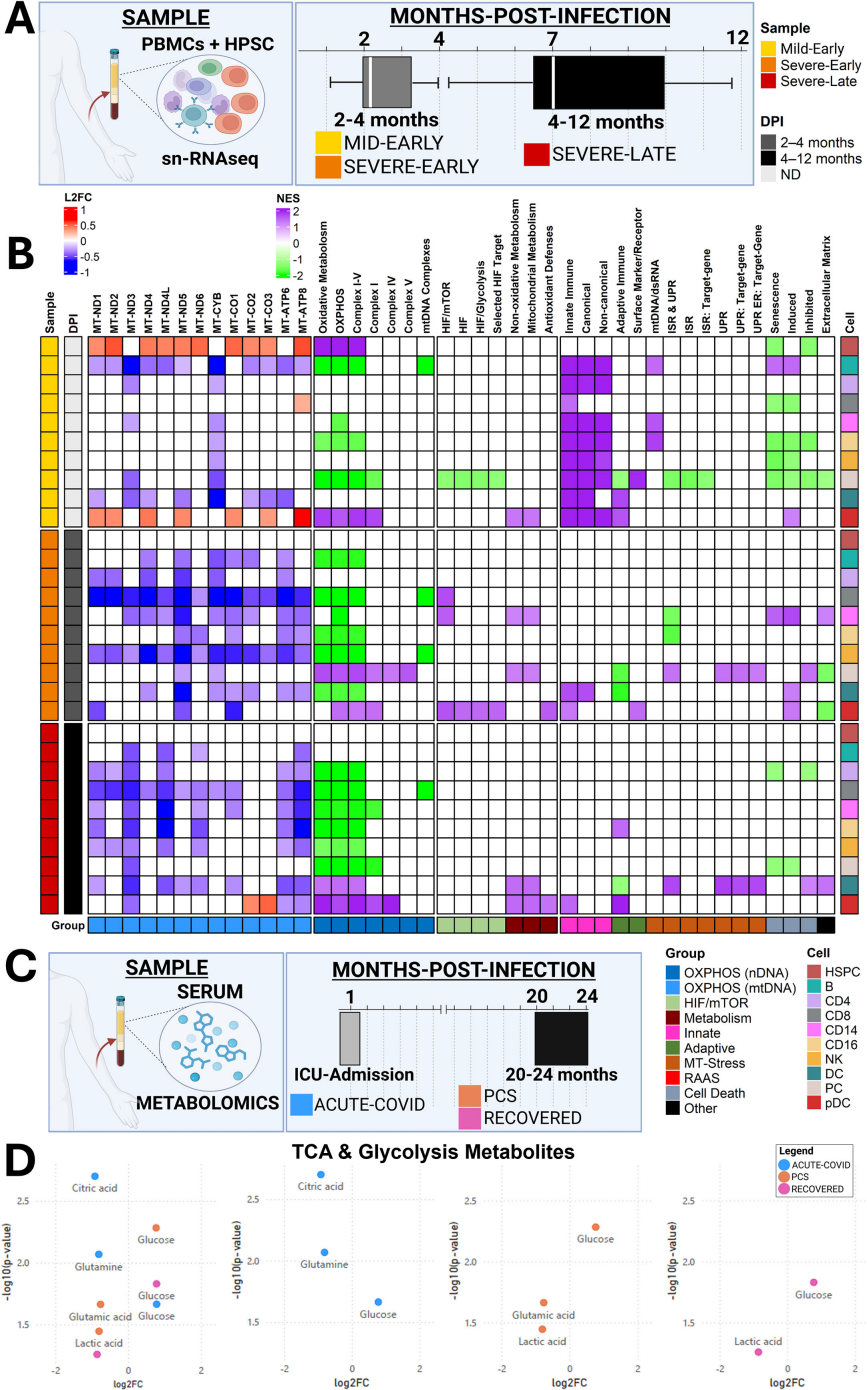
Persistent mitochondrial dysfunction in SARS-CoV-2–infected patient PBMCs and serum up to 1 year post-infection. **(A)** Overview of human PBMC snRNA-seq analysis from mild and severe COVID-19 patients sampled during the early phase (60–120 dpi) or late phase (120–364 dpi). **(B)** Heatmap of NES from GSEA using mitochondrial OXPHOS and immune pathways (FDR threshold = 0.25), and log2 fold change of mitochondrially encoded OXPHOS transcripts (*p < 0.05) in infected vs. uninfected controls. snRNA-seq PBMC dataset **(A, B)**: uninfected healthy controls (n = 7), mild-early (n = 3), severe-early (n = 4), and severe-late (n = 8). Comparisons: mild-early vs. uninfected, severe-early vs. uninfected, and severe-late vs. uninfected. **(C)** Overview of human serum metabolomics datasets analyzed. **(D)** Volcano plot of log10(adjusted p-values) and log2 fold change for glycolytic and TCA cycle metabolites from serum metabolomics. Sample sizes: healthy controls (n = 37), Acute-COVID (n = 14), PCS (n = 31), and Recovered (n = 18). Comparisons: Acute-COVID vs. healthy, PCS vs. healthy, and Recovered vs. healthy. All n values represent biologically independent human subjects.

We next expanded our analysis to longitudinal PBMC datasets collected 2–12 months post-infection using single-cell RNA sequencing (scRNA-seq) (16). Importantly, this cohort was stratified by acute disease severity (mild vs severe requiring ICU care) and time since recovery (early: 2–4 months; late: 4–12 months), rather than by the presence of persistent symptoms (**Figure 5A**). To determine whether mitochondrial and immune dysregulation persists after acute infection, we analyzed PBMCs from mild and severe patients across early and late recovery phases.

Pathway- and transcript-level analyses revealed distinct temporal patterns of mitochondrial regulation across PBMC subtypes (**Figure 5B**). During the early phase, severe cases demonstrated coordinated downregulation of mtDNA-encoded OXPHOS transcripts across complexes I, III, IV, and V in most immune cell types. This suppression was weaker in mild cases, where reductions were more restricted, for example in B cells and dendritic cells. Mild patients also showed partial metabolic recovery, including increased nDNA- and mtDNA-encoded OXPHOS transcripts in plasmacytoid dendritic cells (pDCs) and hematopoietic stem and progenitor cells (HSPCs).

In contrast, late-phase severe patients showed limited evidence of coordinated recovery, instead exhibiting persistent broad suppression of mtDNA-encoded OXPHOS genes across nearly all PBMC subsets, with only isolated exceptions, such as MT-CO2 and MT-CO3. nDNA-encoded OXPHOS pathways were similarly downregulated, indicating sustained nuclear–mitochondrial transcriptional inhibition. Because late-phase samples reflect individuals longitudinally followed after severe disease, these findings suggest that prolonged mitochondrial suppression can persist for months after recovery, independent of symptom stratification, consistent with durable immunometabolic reprogramming described in this cohort (16). Collectively, these results are consistent with the idea that severe SARS-CoV-2 infection is associated with long-lasting repression of mitochondrial bioenergetic programs in circulating immune cells, providing a potential systemic link through which acute disease severity may contribute to prolonged post-acute vulnerability (**Figures 5A, B**).

### Longitudinal serum multi-omics suggest sustained mitochondrial dysfunction and chronic immune-metabolic activation in PCS

#### Metabolomic analysis supports sustained TCA Cycle changes in long-COVID

We next expanded our analysis to human plasma and serum samples collected longitudinally at 1 and 20–24 months-post-infection, via proteomics and metabolomics (16, 43, 44). To investigate systemic metabolic alterations in PCS patients, we next analyzed plasma metabolomic data collected from acutely hospitalized COVID-19 patients early after infection, as well as from COVID-recovered individuals and PCS patients sampled 20–24 months post-infection (44) (**Figure 5C**). Notably, this study (44) observed elevated lactate (via metabolomics) and IL-17 (via biochemical analysis) in PCS patients, consistent with increased glycolytic activity and immune dysfunction.

Our analysis was consistent with these findings. Early-phase samples showed elevated glycolytic intermediates—including glucose and lactic acid—reflecting high metabolic demand, accompanied by decreased TCA cycle intermediates such as glutamic acid, citric acid, and related carbon-flux metabolites. This pattern of increased glycolysis and reduced TCA throughput aligns with the transcriptional evidence of diminished oxidative phosphorylation and is consistent with SARS-CoV-2–associated inhibition of OXPHOS (3, 25–28, 41) (**Figure 5D****;** **Supplementary Figure S5**).

By 20–24 months post-infection, both COVID-recovered and PCS individuals exhibited partial metabolic normalization (**Figure 5D****;** **Supplementary Figure S5D**). Notably, PCS patients retained core metabolic changes from the acute phase, including persistent elevations in glucose and reductions in glutamic acid. Glucose also remained elevated in COVID-recovered individuals, but to a lesser extent, suggesting that SARS-CoV-2 infection exerts lasting metabolic effects even after clinical recovery—though these are more pronounced in PCS patients (**Figure 5D****;** **Supplementary Figure S5C**). Furthermore, both COVID-recovered and PCS patients showed elevated kynurenine, a tryptophan-derived metabolite associated with chronic inflammation, IDO activation, and NAD^+^ pathway remodeling, further linking immune dysregulation to ongoing metabolic strain. These findings suggest that both groups experience persistent mitochondrial metabolic remodeling, characterized by reduced OXPHOS recovery and sustained immune-metabolic activation. The persistence of dysfunction in COVID-recovered patients underscores that metabolic stress and incomplete recovery may be associated with increased vulnerability and potentially predispose susceptible individuals to ongoing post-acute symptoms.

#### Early and late systemic responses diverge in PCS, revealing distinct proteomic signatures associated with pcs progression

**Figure 6:**
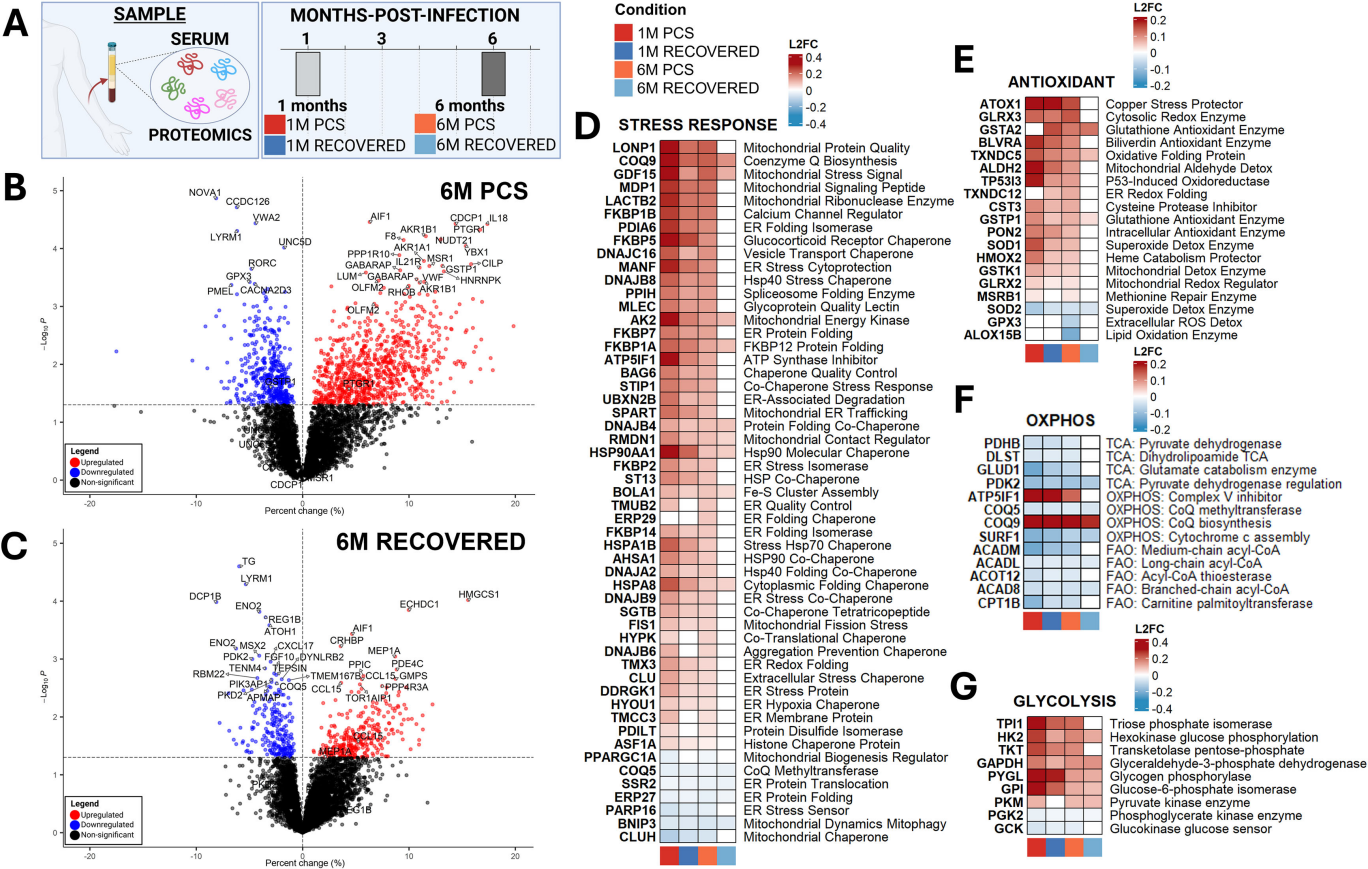
Early and late systemic responses diverge in PCS, revealing unique proteomic signatures of a pathogenic trajectory. **(A)** Overview of human serum proteomic datasets. **(B–C)** Volcano plots of log10(adjusted p-values) and percent changes for patients that went on to develop PCS **(B)** or recovered after SARS-CoV-2-infection **(C)** at 6 months post-infection versus healthy controls. **(D–G)** Heatmaps of Stress Response **(D)**, Antioxidant **(E)**, OXPHOS **(F)**, and Glycolysis **(G)** proteins. P-value threshold = 0.05. Sample sizes for the longitudinal serum proteomics cohort were as follows: healthy-controls (n = 39); 1M PCS (n = 40); 6M PCS (n = 38); 1M Recovered (n = 73); 6M Recovered (n = 75). Comparison: Acute-COVID vs. healthy-controls and Recovered vs healthy-controls. All n values represent biologically independent human subjects.

Building upon the persistent mitochondrial abnormalities identified, we next examined circulating proteomic profiles to determine whether long-term systemic protein expression patterns reflect chronic immune and metabolic dysregulation in PCS patients (**Figure 6A**). In this study (43), patient serum samples were collected at 1 and/or 6 months post-infection for patients tracked for PCS symptom progression at 6 months and 1-year post-infection, enabling the early detection of PCS biomarkers. A key finding was increased complement activation in PCS patients, which we further analyzed to assess mitochondrial-linked immune and metabolic pathways.

Proteomic volcano plots were generated for individuals with PCS and those who had fully recovered at 1- and 6-months post-infection (**Figures 6B, C****;** **Supplementary Figure S6**), followed by pathway-level categorization of differentially expressed proteins (**Figures 6D–G****;** **Supplementary Figure S6**). At 1-month post-infection, individuals who later developed PCS displayed a markedly broader and more pronounced pattern of proteomic dysregulation than those who recovered, encompassing exaggerated induction of innate immune proteins, interferon-stimulated factors, components of the complement cascade, and extracellular matrix-associated proteins (**Supplementary Figure S6**). In contrast, recovered individuals showed a more restricted and resolving antiviral signature, consistent with controlled immune contraction. These early proteomic perturbations in PCS mirror and are consistent with the chronic immune-metabolic signatures observed at 6 months (**Figure 6**), suggesting that PCS may involve the persistence and amplification of an initially maladaptive systemic response, rather than solely the emergence of a late-onset process.

#### Proteomic profiling indicates persistent stress-response and antioxidant upregulation at 6 months in PCS

Compared with recovered individuals, 6-month PCS patients exhibited broad and sustained upregulation of cellular stress-response proteins, particularly chaperones and protein–quality control regulators (**Figure 6D**). These included HSP40/70 co-chaperones (DNAJB and HSPA families), ER-stress and unfolded protein response mediators, and mitochondrial protein-folding and import regulators (CLPP, LONP1). PCS samples also showed elevated levels of GDF-15, a stress-induced mitokine upregulated during mitochondrial dysfunction. Prior studies have consistently linked elevated GDF-15 with COVID-19 severity (26–28, 51–53). This coordinated enrichment is consistent with persistent proteotoxic stress and disrupted protein homeostasis, consistent with chronic mitochondrial dysfunction and unresolved inflammatory signaling.

In parallel, PCS samples also demonstrated continued elevation of antioxidant and redox-buffering enzymes, including superoxide dismutase (SOD1/SOD2) and glutathione-related enzymes (GLRX1/2/3, GSTA2, GSTK1, GSTP1, GPX3), along with TXNDC family proteins, MSRB1, and ALDH-family detoxification enzymes (**Figure 6E**). This antioxidant signature is consistent with ongoing mitochondrial and oxidative stress, aligning with earlier observations of impaired mitochondrial respiration and heightened inflammatory signaling. By contrast, recovered individuals showed minimal or no upregulation of these stress-response or redox-regulating proteins, consistent with resolution of oxidative burden.

The persistence of this oxidative and proteotoxic stress, along with the prolonged elevation, likely contributes to sustained immune dysregulation. In support of these findings, PCS patients exhibit broad, coordinated activation of both innate and adaptive immune protein networks (**Supplementary Figure S7**). On the innate immune side, PCS samples showed marked upregulation of neutrophil-associated chemotactic proteins (CXCL8, CCL7), acute-phase reactants (CRP, CHI3L1), macrophage activation markers (CD163, S100A12, ARG1), interferon-stimulated innate immune mediators (IFI16, IRF3), and complement-related factors (C3, CFD). The sustained presence of these proteins is consistent with ongoing low-grade inflammation, persistent myeloid activation, and unresolved innate immune signaling months after infection. In contrast, recovered individuals displayed attenuation of these pathways, consistent with immunologic resolution.

Similarly, proteins involved in adaptive immune-activation—including cytokine receptors (IL21R, IL2RA), cytotoxic effector molecules (GZMA, GZMB-related pathways), B-cell and T-cell costimulatory/activation markers (CD22, CD79B, LCK, TNFRSF8), and antigen-presentation components (HLA-DRB3, HLA-LA2)—remained chronically upregulated in PCS but not in recovered controls. This persistent enrichment of lymphocyte activation and MHC class II-associated signaling is consistent with continued antigenic stimulation and incomplete immunologic contraction, potentially driven by residual inflammatory cues or ongoing dysregulated antigen presentation.

#### Proteomic signatures of incomplete OXPHOS recovery and glycolytic dysregulation demonstrate metabolic inflexibility in PCS

OXPHOS-associated proteins revealed a clear divergence between PCS and recovered individuals (**Figure 6F**). PCS samples showed mixed or decreased abundance of key mitochondrial metabolic enzymes (PDHB, DLST, ACADM), suggesting persistent inefficiency in TCA flux and fatty acid oxidation. Recovered patients showed a more normalized or upregulated mitochondrial protein profile, indicating improved bioenergetic function. Notably, some PCS samples exhibited upregulation of compensatory mitochondrial proteins (ATP50) consistent with a partial but insufficient attempt to counteract respiratory chain inefficiency.

Long-COVID proteomes demonstrated upregulation of several glycolytic enzymes, such as HK2, TPI1, GAPDH, and PGK1 (**Figure 6G**), suggesting a shift toward glycolytic compensation, potentially driven by impaired mitochondrial ATP production. Recovered patients displayed a more balanced metabolic profile with fewer glycolytic alterations.

Together, these proteomic data are consistent with a model in which 6-month-old PCS is associated with persistent mitochondrial dysfunction (impaired OXPHOS enzyme recovery), chronic oxidative stress (sustained antioxidant protein upregulation), proteotoxic and ER stress activation (upregulated chaperones, UPR mediators), and glycolytic compensation (upregulated glycolytic enzymes). In contrast, individuals who recovered exhibit substantial normalization of mitochondrial, redox, and metabolic protein expression, consistent with restoration of cellular homeostasis.

#### Proposed model: failed mitochondrial recovery contributes to persistent post-acute immune dysregulation

**Figure 7:**
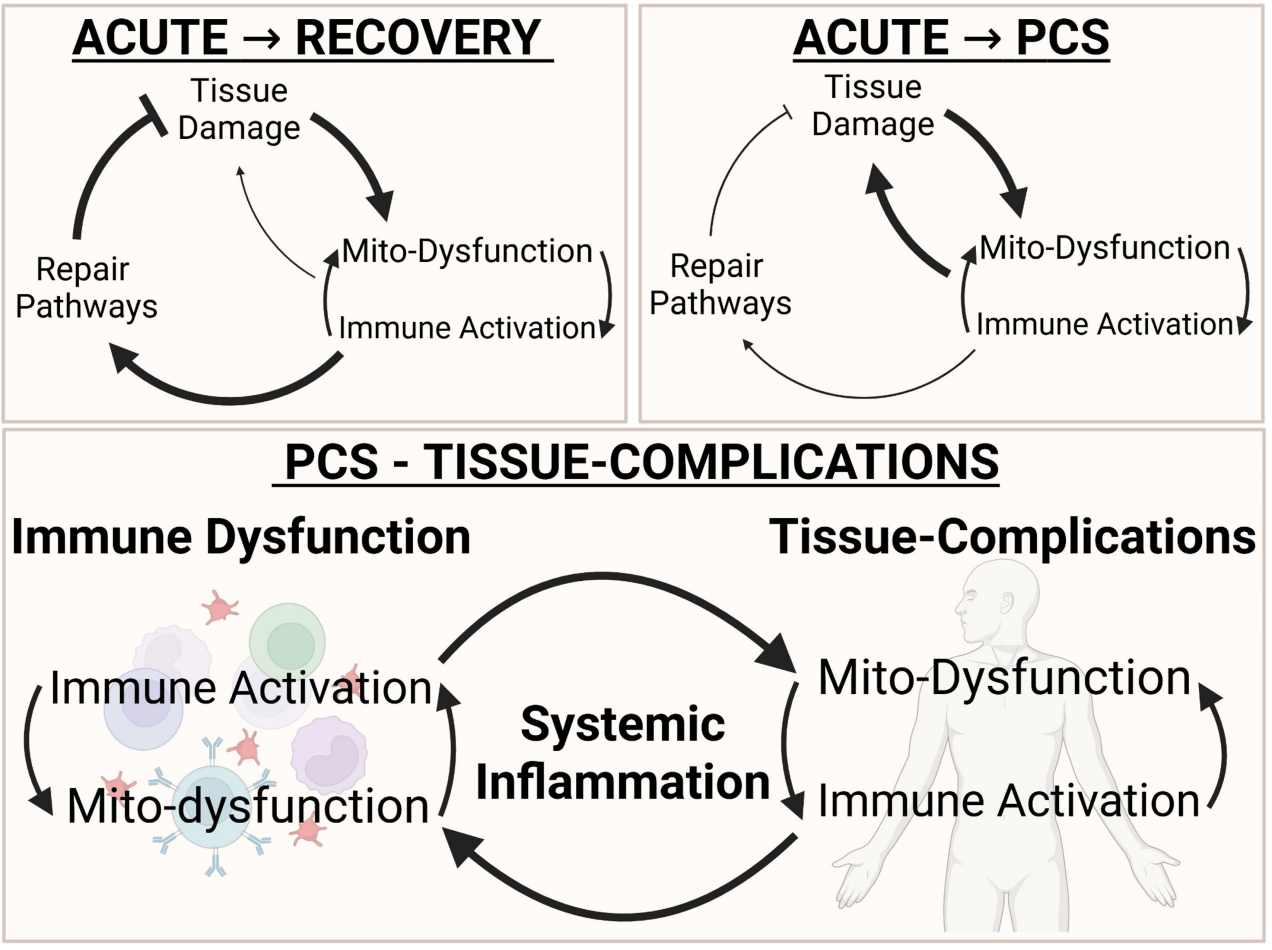
Proposed model: incomplete mitochondrial recovery is associated with persistent post-acute immune dysregulation. Hypothesized framework linking mitochondrial dysfunction with sustained immune activation in PCS. This schematic summarizes an integrated model derived from convergent multi-omics signatures observed across acute severe COVID-19 and PCS-associated datasets. The model proposes a potential feed-forward relationship in which persistent mitochondrial dysfunction may reinforce immune activation and contribute to prolonged post-acute symptoms, whereas recovery is associated with restoration of mitochondrial homeostasis and resolution of inflammatory signaling.

Our findings are consistent with a hypothesized model in which, in healthy patients, mitochondrial repair programs—including antioxidant defenses, mitophagy, and integrated stress responses—may support restoration of bioenergetic and immune homeostasis following infection (**Figure 7**). In contrast, persistent post-acute phenotypes may be associated with sustained mitochondrial dysfunction in one or more tissues, potentially associated with unresolved SARS-CoV-2–induced mitochondrial damage or deficient repair mechanisms. This persistent mitochondrial stress could contribute to prolonged immune activation, consistent with a potential feed-forward relationship between bioenergetic decline and chronic inflammation.

## Discussion

PCS, or Long-COVID, is a chronic, multisystem condition affecting 10–26% of individuals after acute SARS-CoV-2 infection who exhibit persistent symptoms 3 months post-infection. Core symptoms such as memory and concentration deficits, chronic fatigue, muscle weakness, and cardiovascular complications can persist for years post-infection (1–5). Despite its scale, there are no FDA-approved treatments or validated biomarkers for PCS, and the molecular mechanisms linking acute infection to persistent dysfunction remain poorly defined (1, 6, 7).

PCS exhibits multisystemic manifestations that mirror other disorders of mitochondrial dysfunction, involving ME/CFS (5), fibromyalgia, and POTS (1, 3, 4). Following resolution of acute infection, PCS appears to stem from a maladaptive host response that fails to restore immune and metabolic homeostasis, resulting in chronic low-grade inflammation and bioenergetic deficits across high-energy tissues like the brain (8–10), heart (1, 3, 4, 11, 12), skeletal muscle (13–15), and immune system (16–19). This suggests that PCS may arise from complications associated with prolonged mitochondrial dysfunction.

Our previous work and others have mapped the impact of SARS-CoV-2 on host mitochondria, revealing a rapid reprogramming of cellular metabolism during acute illness (3, 25–30, 37), via direct interference with mitochondrial complexes (3, 25, 34), repression of mitochondrial gene transcription (3, 26–28), and stabilizing of HIF-1α and mTOR (3, 26, 27, 39, 40).

In the acute phase, SARS-CoV-2 inhibits OXPHOS to elevate mROS to stabilize HIF-1α. Stabilization of HIF-1α shifts metabolism toward aerobic glycolysis and the pentose phosphate pathway, thereby promoting nucleotide and lipid synthesis, essential for efficient viral replication. Concurrent SARS-CoV-2 induces mTORC1 activation, further reinforcing this Warburg-like metabolic phenotype. SARS-CoV-2-induced mROS production also drives the release of mtDAMPs, amplifying inflammation. Inhibition of these non-oxidative pathways—including glycolysis, the PPP, and HIF/mTORC1 signaling—consistently reduces viral replication, whereas interventions that enhance glycolysis or suppress mitochondrial activity worsen disease (3, 25, 27). Conversely, mitochondria-targeted therapeutics that limit oxidative stress mitigate disease severity in animal models, human monocytes, organoids, and cell cultures, underscoring mitochondrial stress as a central determinant of SARS-CoV-2 virulence and potential target for severe COVID-19 (27, 41).

Beyond the acute phase of infection, mitochondrial dysfunction persists across multiple tissues. Autopsy studies from severe COVID-19 cases revealed long-lasting transcriptomic alterations in the lungs, heart, kidney, liver and lymph nodes, consistent with sustained metabolic stress and incomplete recovery of homeostasis (26, 28). In the autopsy tissues, the heart and kidney exhibited OXPHOS inhibition, HIF-1α activation, and immune upregulation, with the heart also showing upregulation of the RAAS pathway. In the lungs, OXPHOS transcripts, markers of mitochondrial stress, inflammatory cytokines, and the HIF-1α–mTORC1 signaling axis were elevated (26, 28).

Building on this, we observed that human frontal cortex autopsy samples exhibit OXPHOS repression, HIF-1α, immune and RAAS activation, and suppression of neuronal metabolic pathways (**Figure 4**). In parallel, PBMCs from severe COVID-19 patients show marked suppression of mtDNA- and nDNA-encoded OXPHOS transcripts persisting for up to 12 months post-infection (**Figures 5A, B**). Collectively, these findings are consistent with a model in which severe SARS-CoV-2 infection can induce sustained mitochondrial repression across both central and peripheral compartments long after viral clearance. The persistence of this state could contribute to a systemic bioenergetic constraint in which susceptible individuals—particularly those with underlying metabolic dysfunction—may contribute to downstream pathology.

However, important caveats remain. Because these datasets were derived from individuals with severe or fatal COVID-19, they likely reflect the upper bounds of SARS-CoV-2–induced tissue stress and inflammatory activation. Additionally, the PBMC cohort was not stratified based on the presence or absence of persistent symptoms at late timepoints. To investigate if these processes persisted in PCS patients, we employed an integrated multi-omics framework across hamster PCS models, human serum and muscle biopsies.

This hamster model provides a controlled system for studying PCS, as 100% of hamsters develop post-acute SARS-CoV-2 symptoms, compared to the 10–26% prevalence in humans. Although the hamster model does not fully capture the clinical heterogeneity of human PCS, it enables precise investigation of tissue-specific immunometabolic recovery trajectories following SARS-CoV-2 infection experimentally in a short period of time (15, 20). Notably, our transcriptomic profiling of tissues from the PCS hamster model (despite small sample sizes, n=3) recapitulated patterns seen in human autopsy tissues, albeit to a lesser extent, consistent with the reduced pathology observed (**Figures 1**–**4**).

In PCS hamster lungs, OXPHOS expression recovered; however, similar to findings in human lung autopsy samples, mitochondrial stress and immune pathways remained chronically upregulated (**Figure 1**). These profiles correlated with enhanced fibrotic remodeling and reduced respiratory capacity as seen in the prior study (20), offering a mechanistic link between mitochondrial-driven immune tissue scarring and the exertional dyspnea frequently reported in PCS patients (54, 55).

Heart and kidney tissues from PCS hamsters also exhibited persistent mitochondrial impairment, though generally milder than that seen in autopsy samples. Specifically, PCS hamster hearts demonstrated marked OXPHOS repression and sustained immune activation but lacked evidence of RAAS engagement. PCS hamster kidneys displayed moderate OXPHOS inhibition without immune activation, partially mirroring the human pathology (**Figure 1**). Functionally, persistent cardiac mitochondrial dysfunction is consistent with restricted ATP production, that could impair contractility and increase susceptibility to arrhythmogenesis—findings consistent with chest pain, palpitations, and POTS observed clinically (1, 12, 56). The milder mitochondrial deficits observed in the kidneys align with the lower frequency of chronic kidney involvement in PCS, though they may still contribute to episodes of acute renal dysfunction (1).

Among all tissues examined, skeletal muscle exhibited the most profound and persistent mitochondrial repression (**Figure 2**). During acute SARS-CoV-2 infection, hamster skeletal muscle displayed strong immune activation and marked suppression of OXPHOS, which persisted into the PCS phase and was associated with transcriptional signatures of mitochondrial stress and functional decline. Muscle biopsies from PCS patients with chronic fatigue showed similar profiles, including sustained OXPHOS downregulation and ongoing activation of mitochondrial stress pathways even 12 months post-infection—suggesting that impaired skeletal muscle bioenergetics may by a durable feature of PCS. The molecular trajectory we observe—early HIF-1α–driven metabolic reprogramming followed by chronic immune engagement and unresolved mitochondrial stress—mirrors mechanisms described in age-related sarcopenia and T2bFA (57), suggesting that compensatory pathways may ultimately fail to restore metabolic homeostasis.

This apparent lack of mitochondrial recovery may contribute to progressive loss of muscle function and the debilitating fatigue characteristic of PCS. Reduced ATP production within myofibers could limit contractile performance and delay recovery after exertion, potentially promoting post-exertional malaise and the “crash” frequently reported by patients (1, 31). Collectively, these findings support skeletal muscle as a key peripheral tissue in which chronic mitochondrial repression may contribute to systemic PCS symptoms, while parallel mitochondrial and inflammatory alterations in the heart and lungs may further contribute to dyspnea, palpitations, and reduced exercise tolerance in affected individuals.

In addition to peripheral tissues, the nervous system displayed striking yet distinct metabolic and immunological trajectories (**Figures 3**, **4**). Neurological complications are among the most frequent and persistent outcomes of PCS, manifesting as cognitive impairment (“brain fog”), unrefreshing sleep, and prolonged sensory deficits such as anosmia/dysgeusia (5, 7, 31, 58). Neuroimaging and post-mortem analyses reveal structural and functional brain alterations, including cortical thinning, decreased subcortical nuclear volume (47), anterior cingulate cortex atrophy (48), and increased BBB permeability (9), accompanied by microglial activation (49), accumulation of senescent cells, and secretion of SASP factors (21, 22). In this context, our results demonstrate that mitochondrial injury does not follow a uniform course across the brain but instead diverges along discrete anatomical trajectories.

Sensory regions such as the olfactory bulb and epithelium, both positioned outside the protection of the BBB, exhibited robust acute inflammation that persisted into the PCS phase and was accompanied by OXPHOS inhibition and transcriptional signatures of mitochondrial stress and senescence (**Figure 3**). Nevertheless, these regions showed partial metabolic normalization over time, consistent with the gradual recovery of smell and taste reported clinically (59). In contrast, deeper brain structures, including the cortex, striatum, and thalamus, displayed acute immune activation and OXPHOS suppression but exhibited heterogeneous recovery, with the frontal cortex emerging as uniquely vulnerable (**Figure 4**). PCS-phase hamster cortex demonstrated persistent OXPHOS repression, impaired neuronal metabolic pathways, consistent with the cognitive heterogeneity and long-lasting executive dysfunction associated with PCS (50). The cerebellum displayed yet another distinct profile characterized by transcriptional upregulation of OXPHOS genes but reduced functional output, suggesting compensatory metabolic rewiring that ultimately fails to restore cellular homeostasis. Taken together, these findings suggest that PCS-associated neurological symptoms may reflect heterogeneous neuroimmune and metabolic alterations driven by systemic inflammation. We propose that differential cytokine exposure, intrinsic bioenergetic vulnerability, and blood–brain barrier integrity could contribute to region-specific susceptibility, although direct CNS profiling will be required to test this hypothesis.

Extending beyond the nervous system, metabolomic and proteomic analyses of human serum samples revealed pervasive systemic immune dysfunction and mitochondrial impairment in PCS patients (**Figures 5**, **6**). Metabolic signatures showed elevated mitochondrial stress markers and elevated glycolytic intermediates, consistent with metabolic reprogramming away from oxidative metabolism (**Figures 5B, C****;** **Supplementary Figure S5**). Proteomic serum profiling demonstrated early divergence between recovered individuals and those with persistent post-acute symptoms, with unresolved inflammatory and metabolic stress signatures detectable at 1 month-post-infection and persisting up to 6 months in PCS patients (**Figures 5**-**6****;** **Supplementary Figures S6**-**7**). Proteomic analyses further revealed sustained activation of mitochondrial stress-response pathways and elevated levels of innate and adaptive immune proteins (**Figure 6****;** **Supplementary Figures S5**–**7**). These stress-response regulators and mitochondrial proteostasis factors formed central hubs within PCS-specific protein networks, indicating ongoing proteotoxic and mitochondrial stress rather than resolution toward homeostasis. While recovered individuals restored immune balance, PCS patients showed continued hyperactivation of both innate and adaptive immunity, reflecting chronic systemic inflammation with multisystem clinical consequences.

These findings also suggest potential translational opportunities through repurposing existing immunomodulatory and metabolic therapies. For example, glucagon-like peptide-1 (GLP-1) receptor agonists, widely used for obesity, have demonstrated anti-inflammatory and mitochondrial-protective properties, including improved efficiency of ATP production, inhibition of apoptosis, and immunomodulatory effects (60). GLP-1 receptor agonists can cross the blood-brain barrier and exhibit neuroprotective effects in multiple disease contexts, raising the possibility that they may modulate persistent metabolic and inflammatory disturbances seen in PCS (61).

In addition, senescence-targeting strategies warrant further investigation. Senolytic agents have been shown to reduce senescent cell burden and associated inflammatory secretory phenotypes in fibrotic and aging-related diseases (62, 63). As senescent cells exhibit both pro-inflammatory signaling and mitochondrial dysfunction, targeting cellular senescence could represent another strategy to potentially modulate the cycle of mitochondrial stress and immune activation identified in PCS. These therapeutic implications remain speculative and will require experimental validation in future studies. Future work should therefore evaluate whether restoring mitochondrial function or modulating senescence pathways can disrupt the immunometabolic feedback loop proposed here.

The datasets integrated in this study span multiple tissues, disease severities, time points, and experimental platforms, and were generated from distinct cohorts rather than the same individuals. Therefore, while convergent patterns identified here are reproducible across datasets, they do not necessarily indicate that all molecular features co-occur within the same cells or patients. Rather, our integrative framework reveals recurrent immunometabolic themes that independently emerge across diverse biological contexts following SARS-CoV-2 infection. The consistency of mitochondrial dysfunction and immune dysfunction across these heterogeneous datasets supports their relevance to PCS complications, even as the precise temporal relationships and cellular co-localization of these features remain unresolved.

### Convergent multi-omics highlight mitochondrial dysfunction in PCS

Collectively, our data supports a model in which mitochondrial dysfunction may contribute to persistent post-acute symptoms (**Figure 7**). SARS-CoV-2 infection induces mitochondrial dysfunction, particularly in energy-demanding tissues, which can persist long after viral clearance (3, 25–30, 37). In individuals who recover, mitochondrial quality control mechanisms—including antioxidant defenses, mitochondrial turnover, and stress-response pathways—may restore bioenergetic homeostasis and support resolution of immune activation. By contrast, persistent symptoms may result from impaired mitochondrial repair or ongoing mitochondrial injury, potentially establishing a feed-forward cycle in which mitochondrial dysfunction further amplifies inflammatory signaling and immune activation further perpetuates mitochondrial stress. This cycle may be more pronounced in those with underlying metabolic disease, lowering the threshold for chronic tissue stress and delaying recovery.

Together, this framework highlights mitochondrial dysfunction as a plausible contributor to post-acute disease biology and suggests that therapeutic strategies aimed at restoring mitochondrial integrity, reducing oxidative stress, and promoting mitochondrial biogenesis could help attenuate this cycle and support recovery.

### Study limitations

Several limitations should be considered when interpreting these findings. First, the analyses are observational and rely on re-analyzing published transcriptomic, proteomic, and metabolomic datasets from various cohorts. Although the convergence of mitochondrial and immune dysregulation across tissues is compelling, these data cannot establish causality or determine whether mitochondrial dysfunction drives PCS or is a consequence of persistent inflammation.

Second, both human and animal datasets have important translational limitations. Some human datasets, particularly those from brain transcriptomes, were derived from acute or fatal COVID-19 autopsy tissues, which likely reflect end-stage disease. These are heavily influenced by critical illness factors, including prolonged hypoxia, mechanical ventilation, systemic shock, and inflammatory injuries associated with ICUs. Therefore, the transcriptional signatures observed may not be directly relevant to the pathogenesis of PASC, as most PASC patients do not experience critical illness. Additionally, several longitudinal datasets of PBMC are stratified by acute disease severity and recovery time rather than persistent symptom status, making it difficult to determine whether the observed long-term mitochondrial suppression is specific to PASC or reflects broader post-viral recovery.

Similarly, while the Syrian hamster model provides a valuable controlled system for studying post-acute molecular trajectories, nearly 100% of infected hamsters develop PCS-like phenotypes compared to only about 10-26% of humans with PASC/PCS. This suggests that the hamster model may represent an exaggerated post-viral phenotype that fails to capture the heterogeneity, risk factors and incomplete penetrance characteristic of human PASC. Furthermore, hamster datasets were generated with small sample sizes (typically n=3 per timepoint), which limits statistical power and increases susceptibility to biological variability.

Thus, while this multi-omics framework indicates shared immunometabolic disruption, these limitations underscore the need for larger, prospective studies with direct PASC phenotyping and comprehensive multi-tissue profiling to clarify causal mechanisms and therapeutic relevance.

## Resource availability

### Lead contact

Further information and requests for resources, data and code availability should be directed to the corresponding authors Joseph W. Guarnieri (joseph.guarnieri@bmsis.org) and Elizabeth Aslinger (elizabeth.aslinger@aya.yale.edu).

### Materials availability

This study did not generate new, unique reagents or materials. All datasets analyzed in this research were obtained from publicly accessible datasets. The specific references and dataset accession numbers are listed in the “Experimental Model and Subject Details” below. Custom pathways used for gene set analysis are described in detail in “Methods Details” below.

### Data and code availability

All data used in this study are publicly available and can be accessed through their respective repository using the accession numbers listed in the “Experimental Model and Subject Details” section below. Code and gene lists utilized in this paper, can be found at https://github.com/easlinger, and https://github.com/shehbeel. Any additional information required to reanalyze the data reported in this paper is available from the lead contact upon request.

## Experimental model and subject details

### Overview

This manuscript integrates data from a diverse set of experimental models spanning animal, tissue, blood-based, and postmortem human studies. Each dataset was originally generated by prior investigators and is described in full detail within the corresponding publications; readers are referred to those citations for comprehensive methodological information (14–16, 20, 22, 42–44).

In brief, data extracted from each source are summarized as follows. RNA-seq datasets: acute and PCS hamster models for skeletal muscle [(15); GSE231910]; heart, liver, lung, and multiple brain regions [(20); GSE203001]; human skeletal muscle PCS and T2bFA [(14); in Supplemental]; and human COVID-19 autopsy frontal cortex [(42); GSE188847] and substantia nigra [(22); GSE174745] samples. snRNA-seq datasets: PBMCs from mild and severe COVID-19 patients (16; GSE196990). Proteomics: PCS and recovered serum samples [(43); in Supplemental]. Metabolomics: acute, PCS, and recovered serum samples [(44); https://data.mendeley.com/datasets/gc9g2g53kr/1].

Below, we also provide a concise summary of the studies and sample types analyzed in this paper for comparative multi-omics integration.

### Hamster model for the heart, lung, kidney, muscle, and brain tissues

The Golden Syrian hamster model recapitulates key aspects of human COVID-19, including high viral replication in the respiratory tract, strong systemic interferon responses, and organ-specific inflammatory signatures that persist into chronic stages (15, 20). Longitudinal studies have demonstrated that SARS-CoV-2-infected hamsters develop sustained, multi-organ transcriptional and inflammatory alterations after recovery that parallel molecular features observed in humans following acute infection. In the present study, we leverage this validated model to examine post-acute immunometabolic remodeling across tissues. While hamsters do not model the full heterogeneity of PCS as defined in humans, they provide a mechanistically tractable system for investigating persistent molecular consequences of SARS-CoV-2 infection.

For the hamster tissues analyzed in this study, 6- to 7-week-old male Golden Syrian hamsters (Mesocricetus auratus) were obtained from Charles River Laboratories and acclimated in a CDC/USDA-approved BSL-3 facility for at least 7 days prior to infection. Animals were intranasally inoculated under ketamine/xylazine anesthesia with either phosphate-buffered saline (mock) or SARS-CoV-2 USA-WA1/2020 (1×10^3^ PFU in 100 μL). Hamsters were euthanized at 3, 31, and—specifically for skeletal muscle analyses—61dpi. Following PBS perfusion, lung, heart, kidney, quadriceps muscle, and multiple brain regions—including the olfactory bulb, medial prefrontal cortex, striatum, thalamus, cerebellum, and trigeminal ganglion—were harvested. Tissues designated for viral quantification and transcriptomic analyses were homogenized in PBS or TRIzol. Infectious viral titers in lung tissue were quantified via plaque assay on Vero-E6 cells, while SARS-CoV-2 RNA, including subgenomic N, was measured by qRT-PCR (15, 20).

In addition to transcriptomic analyses, behavioral and biochemical assays were performed on the infected hamsters. Notably, histology-associated inflammation was observed in the lungs and kidneys, along with behavioral and molecular changes consistent with SARS-CoV-2–related brain dysfunction (20). Furthermore, quadriceps muscle displayed signatures of structural, inflammatory, and microvascular alterations, including capillary remodeling, macrophage infiltration, interferon-driven responses, and complement activation (15).

### Human model for the vastus lateralis muscle tissue

Human skeletal muscle samples were obtained from the vastus lateralis of individuals with a history of COVID-19, including patients with ongoing post-COVID symptoms (PCS), alongside healthy SARS-CoV-2–negative controls (14). The study also included two disease-based control cohorts: a healthy-diseased control (HDC) group of clinically symptomatic individuals with histologically normal biopsies, and a type-2B–fiber atrophy (T2BFA) cohort, which exhibited selective T2BFA without other muscle pathology. Muscle biopsies were collected percutaneously under local anesthesia and immediately processed, with portions flash-frozen for transcriptomic and biochemical analyses and others fixed in 4% paraformaldehyde for histopathology and immunohistochemistry. RNA sequencing was performed to assess transcriptional changes, comparing PCS and T2BFA to their respective healthy controls. PCS and T2BFA tissues displayed features consistent with their clinical conditions, including Type II fiber atrophy, altered capillary density, macrophage infiltration, and molecular signatures of interferon signaling and complement activation (14).

### Human model for serum metabolomics

Serum metabolomics analysis was performed using targeted quantitative LC–MS/MS and FIA–MS/MS profiling of 108 metabolites, following protocols established for long-COVID cohort studies (44). Plasma samples were collected from healthy SARS-CoV-2–negative controls, individuals with confirmed acute COVID-19, and patients evaluated approximately 2 years (20–24 months) post-infection for persistent post-COVID symptoms. All participants were recruited between 2020 and 2022 and provided fasting blood samples processed immediately for metabolite extraction. Targeted metabolomics quantified amino acids, acylcarnitines, phospholipids, sphingomyelins, organic acids, and key redox/energy metabolites. Key alterations included changes in lactate/pyruvate and ornithine/citrulline ratios, arginine metabolism, and lipid species, as well as elevated lactate (via metabolomics) and IL-17 (via biochemical analysis) in a subset of long-COVID patients (44).

### Human model for serum proteomic analysis

Serum proteomics was performed on longitudinal blood samples collected from healthy SARS-CoV-2–negative controls and PCR-confirmed COVID-19 patients followed for up to 12 months after infection (43). Proteomic profiling quantified more than 6500 serum proteins using the SomaScan aptamer-based platform. Blood samples were collected during acute infection and again at approximately 1- and 6-months post-infection, enabling analysis across four clinical states: PCS (long-COVID) at 1 month, PCS at 6 months, COVID-recovered at 1 month, and COVID-recovered at 6 months, with healthy controls serving as the reference group. Patients were classified according to symptom persistence at each timepoint, allowing discrimination between early versus unresolved post-acute abnormalities (43).

### Human model for PBMCs+HPSCs snRNA-seq analysis

PBMCs and circulating HSPCs were analyzed using approaches adapted from Cheong et al., 2023 (16). Blood was collected from three clinical groups: healthy SARS-CoV-2–negative controls and early convalescent severe COVID-19 (2–4 months post-onset), and late convalescent severe COVID-19 (4–12 months post-onset). Participants were grouped by acute disease severity (severe COVID-19 requiring ICU-level care; WHO score 6–7) and time since recovery (early: 2–4 months; late: 4–12 months), and were not stratified by persistent symptom status at late timepoints. PBMCs were isolated by density-gradient centrifugation, and rare circulating CD34^+^ HSPCs (~0.05% of PBMCs) were enriched using magnetic separation. Enriched PBMC–HSPC suspensions were subjected to multimodal single-cell profiling using combined single-nuclei RNA-sequencing and ATAC-sequencing (snRNA/ATAC-seq) (16).

### Autopsy datasets and ICU cohorts

Autopsy datasets reflect end-state disease following severe acute SARS-CoV-2 infection and were included to characterize the maximal extent of tissue-level immunometabolic perturbation, rather than to model post-acute sequelae of COVID-19. Cohorts requiring ICU-level care were analyzed separately, with the recognition that critical illness, mechanical ventilation, and prolonged hospitalization are independently associated with long-term immunometabolic alterations. Cohorts analyzed in this study include those summarized in **Figure 1A**, highlighting major pathway changes (20, 26), as well as autopsy tissues from the frontal cortex (42) and substantia nigra (22).

### Human model for the frontal Cortex RNA-seq analysis

Postmortem frontal cortex (Brodmann area 8) was collected from individuals with severe COVID-19, age- and sex-matched uninfected controls, and an ICU/ventilator-treated uninfected cohort, with COVID-19 status confirmed by pre-/peri-mortem SARS-CoV-2 qPCR (42). Frozen tissue was processed under BSL-2+ conditions, homogenized in TRIzol, and total RNA was extracted and prepared using standard ribodepletion-based RNA-seq.

### Human model for the substantia Nigra RNA-seq analysis

Postmortem substantia nigra tissue was obtained from individuals with PCR-confirmed COVID-19 and age- and sex-matched uninfected controls through the NIH NeuroBioBank and affiliated repositories (22). Tissue identity and dopaminergic neuron content were verified using NR4A2 immunostaining, and COVID-19 status was confirmed by clinical documentation and SARS-CoV-2 testing. Frozen substantia nigra samples were homogenized in TRIzol, and total RNA was extracted and processed for RNA-seq using standard library preparation workflows.

## Method details

### Data acquisition and processing (RNA-seq, snRNA-seq)

All bulk RNA-seq and snRNA-seq datasets were obtained from previously published studies, with library preparation, sequencing, and initial preprocessing described in the original sources (Experimental Model and Subject Details for more details) (14–16, 20, 22, 42). Bulk RNA-seq data were processed using standard alignment and quantification pipelines to generate raw gene-level count matrices. For scRNA-seq datasets, author-provided gene–barcode matrices were used as inputs for downstream analysis.

Bulk RNA-seq count tables were imported into DESeq2 (v1.46.0) (64), and snRNA-seq matrices were preprocessed according to their respective publications before pseudo-bulk conversion. Count matrices from all RNA-seq modalities were assembled into DESeqDataSet objects with defined experimental designs. After internal size-factor normalization, DESeq2 fit negative binomial generalized linear models and computed all pairwise contrasts, yielding normalized counts, log2 fold changes, standard errors, Wald statistics, p-values, and FDR-adjusted p-values. Gene annotations were appended using AnnotationDbi (v1.66.0), org.Hs.eg.db (v3.18.0) or org.Mm.eg.db (v3.18.0), and online retrieval via biomaRt (v2.58.0) when needed.

Gene-level results were used for visualization, pathway scoring, and enrichment analyses. Volcano plots were generated with EnhancedVolcano (v1.14.0) (65); heatmaps with ComplexHeatmap (v2.9.4) (58) and circlize (v0.4.12); and lollipop plots using ggplot2 (v3.5.0) for plotting, ggh4x (v0.2.5) for facet_grid2 layouts, and scales (v1.3.0) for gradient and size transformations, with grDevices used internally for color handling.

### Metabolomic data processing and differential abundance analysis

Targeted LC–MS/MS and FIA–MS/MS metabolomic datasets were preprocessed and normalized according to their source publications (44). Differential metabolite abundance was analyzed using limma (v3.52.4) (66), with a design matrix constructed from sample metadata and linear models fitted for each metabolite. Empirical Bayes moderation—with trend and robust options—was applied to stabilize variance estimates. Pairwise contrasts were computed to identify alterations in key metabolic pathways, including energy metabolism, amino acid and nucleotide metabolism, lipid metabolism, and redox balance. Resulting log_2_ fold changes, t-statistics, p-values, and FDR-adjusted p-values were compiled, and graphs were generated in PowerBI to visualize metabolite- and pathway-level changes.

### Proteomic sample preparation and differential analysis

Protein abundance was quantified using the SomaScan aptamer platform, measuring over 6,500 serum proteins according to their source publications (43). Expression matrices were generated from raw seq.* identifiers, and a limma (v3.52.4) (66) linear modeling framework was applied with design matrices derived from clinical groupings (Healthy, Acute-COVID, Recovered, PCS). Empirical Bayes moderation stabilized variance estimates, and all pairwise contrasts were computed to identify protein-level changes associated with viral infection, recovery, and persistent post-COVID symptoms. Seq.* identifiers were mapped to gene symbols and UniProt IDs, then cross-referenced to official HGNC symbols using biomaRt (v2.58.0) for downstream protein-level analysis and visualization. Volcano plots were generated with EnhancedVolcano (v1.14.0) (65); heatmaps with ComplexHeatmap (v2.9.4) (58) and circlize (v0.4.12).

### Custom pathways

Custom muscle-related pathway lists were generated by combining pre-established gene sets from the Gene Ontology Biological Process (GOBP), Human Phenotype Ontology (HP), Reactome Pathway Database (Reactome), Gene Ontology Molecular Function (GOMF), Gene Ontology Cellular Component (GOCC), and the WikiPathways Database (WikiPathways). These pathways were organized into six macro-categories: “MUSCLE”; “SKELETAL MUSCLE”; “STRIATED MUSCLE”; “MUSCLE WEAKNESS”; “ABNORMAL”; “PATHOLOGY”. To facilitate clustering and interpretation, we assigned simplified names to these pathways for use in this study. Pathway sources, original pathway names, and the names applied here for each pathway macro-category are provided in **Supplementary Table S1**.

Custom brain-related pathway lists were generated by combining pre-established Gene Ontology pathways and organizing them into three macro-categories: “Behavior & Cognition”; “Neuronal Function”; and “Synapse.” As with the muscle pathways, we assigned simplified names to facilitate more apparent clustering and interpretation. Pathway sources, original GO pathway names and GO ID numbers, as well as our assigned simplified names for each macro-category, are detailed in **Supplementary Table S1**. In addition, several new pathways were created by merging multiple GO gene lists and removing duplicates, as also specified.

To examine extracellular matrix (ECM) remodeling, we evaluated genes associated with the *Extracellular Matrix Organization* pathway as defined in the Reactome database (Reactome ID: R-HSA-1474244). For senescence-related genes, we curated “Senescence,” “Induced Senescence,” and “Inhibited Senescence-associated genes were compiled from the Database of Cell Senescence Genes (https://genomics.senescence.info/cells/).

All remaining pathways used in this study were derived from previously published custom metabolic and immune gene sets (26, 28), with a few minor changes. For the metabolic pathways (26) related to mitochondrial biology, we incorporated the custom OXPHOS-focused gene lists and consolidated all mitochondrial OXPHOS-related pathways—including “Complex I–V,” “Complex I,” “Complex II,” “Complex III,” “Complex IV,” “MT-Ribosome,” “MT-Biogenesis,” “MT-Protein Import,” and “MT-CoQ Synthesis”—into a newly defined macro-category termed “OXPHOS.” For glycolytic and hypoxia-responsive pathways, we created an additional macro-category, “HIF/mTOR,” that integrates the “HIF1A,” “mTOR,” and “Glycolysis” pathways. Finally, from the custom immune pathway set (27), we established two broader categories to capture integrated stress and cell-death responses: “ISR & UPR,” which merges the integrated stress response and unfolded protein response pathways, and “PANoptosis,” which unifies the “Pyroptosis”, “Necroptosis”, and “Apoptosis” programs into a single composite pathway.

### Figure preparation

Summary schematics and conceptual illustrations were created using BioRender (BioRender.com). BioRender was used exclusively for graphical representation and did not involve data manipulation or quantitative processing. Final figure layouts were assembled using standard vector graphic software.

## Quantification and statistical analysis

All statistical analyses were performed in R (v4.3.0) using modality-appropriate packages. For transcriptomic analyses, DESeq2 (v1.46.0) was used for normalization and differential expression testing, with gene annotations obtained from AnnotationDbi (v1.66.0), org.Hs.eg.db (v3.18.0) or org.Mm.eg.db (v3.18.0), and online retrieval via biomaRt (v2.58.0) when needed. Proteomic and metabolomic differential abundance analyses were performed using limma (v3.52.4) (66). Unless otherwise specified, p-values were adjusted using the Benjamini–Hochberg method. For pathway-level analyses, an adjusted FDR < 0.25 threshold was applied for significance across all GSEA and gene set enrichment outputs. For visualizations displaying individual metabolites, proteins, or transcripts—including volcano plots, lollipop plots, and heatmaps—features meeting a threshold of p < 0.05 were considered significant.

Figures and data visualizations were generated using EnhancedVolcano (v1.14.0) (65) for volcano plots, ComplexHeatmap (v2.9.4) (67) and circlize (v0.4.12) for heatmaps, ggplot2 (v3.5.0) for plotting, ggh4x (v0.2.5) for advanced facet layouts, and scales (v1.3.0) for gradient and size transformations. Additional data handling and I/O packages included readxl and openxlsx for Excel import/export, and reshape2 for data reshaping. Metabolomic data and pathway-level changes were additionally visualized using Power BI for interactive figures and summary graphs.

## Data Availability

The original contributions presented in the study are included in the article/**Supplementary Material**. Further inquiries can be directed to the corresponding author.
