# Supplementary material for: Multi-omics analysis of long COVID (post-COVID-19 condition) reveals persistent mitochondrial dysfunction, suppressed oxidative phosphorylation, and immune dysregulation
Source: Front Immunol. 2026 May 21;17:1776555. doi: 10.3389/fimmu.2026.1776555 (PMC13234542; doi:10.3389/fimmu.2026.1776555)
Supplement: Supplementary file 1 [file SupplementaryFile1.docx]

**Supplemental Information**

**A multi-omics signature of bioenergetic failure and immune dysregulation defines long-COVID.**

Alexia Tasoula^1,2^, Shehbeel Arif^1,3^, Ethan Waisburg^4^, Lucas Bauer^1,5^, Elizabeth Aslinger^6^*, Joseph W. Guarnieri^1,7^*

**Table of Contents**

**Table S1. Muscle and Brain custom pathway lists, sources and names, and renamed pathway labels for all custom pathway macro-categories** ………………….………..………..……………………………..………..…1

**Figure S1. Dot plots of OXPHOS transcripts in heart, kidney, and lung tissues in a hamster model of PCS.**………………………….………………………………………………………………..…………………….………4

**Figure S2. Dot plots of OXPHOS transcripts in skeletal muscle from human and hamster PCS models**.…………………………………………………………….……………………………………………….………5

**Figure S3. Dot plots of OXPHOS transcripts in sensory brain regions in a hamster model of PCS**.…………………………………………………………………...…………………………………………….………6

**Figure S4. Dot plots of OXPHOS transcripts in cognitive brain regions from human COVID-19 autopsy tissues and a hamster PCS model**.…………………………………………………….………………………………7

**Figure S5. Volcano plots of significant serum metabolic signatures in acute-COVID-19, and in PCS and recovered patients 2 years post-infection**…...……………………………………………………………….………8

**Figure S6. Volcano plots of serum proteomic signatures in PCS and recovered patients at 1 month post-infection**...………………………………………………….………………………………………...….….…………….10

**Figure S7. Heatmaps of serum proteomic innate and adaptive immune signatures in PCS and recovered patients at 1 and 6 months post-infection**......……………………………...…………….....................................11

**Table S1. Muscle and Brain custom pathway lists, sources and names, and renamed pathway labels for all custom pathway macro-categories.**

**Table S1 (continued). Muscle and Brain custom pathway lists, sources and names, and renamed pathway labels for all custom pathway macro-categories.**

**Table S1 (continued). Muscle and Brain custom pathway lists, sources and names, and renamed pathway labels for all custom pathway macro-categories.** Custom pathway lists for muscle and brain analyses. Muscle-related pathways were compiled from GOBP, HP, Reactome, GOMF, GOCC, and WikiPathways and grouped into six macro-categories: Muscle, Skeletal Muscle, Striated Muscle, Muscle Weakness, Abnormal, and Pathology. Brain-related pathways were generated from Gene Ontology and organized into three macro-categories: Behavior & Cognition, Neuronal Function, and Synapse. Simplified pathway names were assigned to facilitate clustering and interpretation. Pathway sources, original pathway names, GO IDs, and assigned names are provided in Table S1; additional pathways created by merging GO gene lists with duplicates removed are indicated.

**Figure S1.** **Dot plots of OXPHOS transcripts in heart, kidney, and lung tissues in a hamster model of PCS.** Dot plots of stat (t-stat) values of OXPHOS transcripts in RNA-seq datasets from hamster lung, kidney, and heart samples. RNA-seq sample sizes for heart, kidney and lung tissues were as follows: Acute-phase (3 dpi): SARS-CoV-2-infected (n=3), Mock (n = 4). Post-acute phase (31 dpi): SARS-CoV-2-infected (n=3), Mock (n = 3). GSEA comparisons: SARS-CoV-2–infected vs. Mock. All n values represent biologically independent animals.

**Figure S2.** **Dot plots of OXPHOS transcripts in skeletal muscle from human and hamster PCS models.** Dot plots of stat (t-stat) values of OXPHOS transcripts in RNA-seq datasets from human and hamster skeletal muscle samples. RNA-seq samples were as follows: Hamster skeletal muscle: SARS-CoV-2–infected at 3 dpi (n = 3), 31 dpi (n = 3), and 61 dpi (n = 3), Mock (n = 3). GSEA comparisons: SARS-CoV-2–Infected vs. Mock. Human skeletal muscle: PCS patients (n = 11), Type IIb fibers atrophy patients (T2bFA) (n = 8), Control Patients (n = 8). GSEA comparisons: PCS vs. Control and T2bFA vs. Control. All n values represent biologically independent samples.

**Figure S3. Dot plots of OXPHOS transcripts in sensory brain regions in a hamster model of PCS.** Dot plots of stat (t-stat) values of OXPHOS transcripts in RNA-seq datasets from hamster sensory brain regions (TG = Trigeminal Ganglia, OB = Olfactory Bulb, OE = Olfactory Epithelium). RNA-seq samples were as follows: OB: SARS-CoV-2-infected 3 dpi (n = 3), Mock 3 dpi (n = 3), SARS 31 dpi (n = 3), Mock 31 dpi (n = 3). OE: SARS 31 dpi (n = 4), Mock 31 dpi (n = 4). TG: SARS 3 dpi (n = 3), Mock 3 dpi (n = 3), SARS 31 dpi (n = 5), Mock 31 dpi (n = 5). GSEA comparisons: SARS-CoV-2–Infected vs. Mock. All n values represent biologically independent animals.

**Figure S4.** **Dot plots of OXPHOS transcripts in cognitive brain regions from human COVID-19 autopsy tissues and a hamster PCS model.** Dot plots of stat (t-stat) values of OXPHOS transcripts in RNA-seq datasets from human and hamster cognitive brain regions. RNA-seq sample sizes were as follows. Human brain tissues: Frontal cortex (FC): COVID-19 autopsy (n = 20) and non-COVID autopsy controls (n = 22); substantia nigra (SN): COVID-19 autopsy (n = 6) and non-COVID autopsy controls (n = 3). GSEA comparisons: COVID-19 autopsy vs. non-COVID autopsy controls. Hamster brain regions: 3 dpi groups included mPFC, striatum, thalamus, and cerebellum with SARS-CoV-2–infected (n = 3) and Mock (n = 3). 31 dpi groups included SARS-CoV-2–infected mPFC (n = 3), striatum (n = 4), thalamus (n = 3), and cerebellum (n = 5), with Mock (n = 5). GSEA comparisons: SARS-CoV-2–infected vs. Mock. All n values represent biologically independent animals.

**
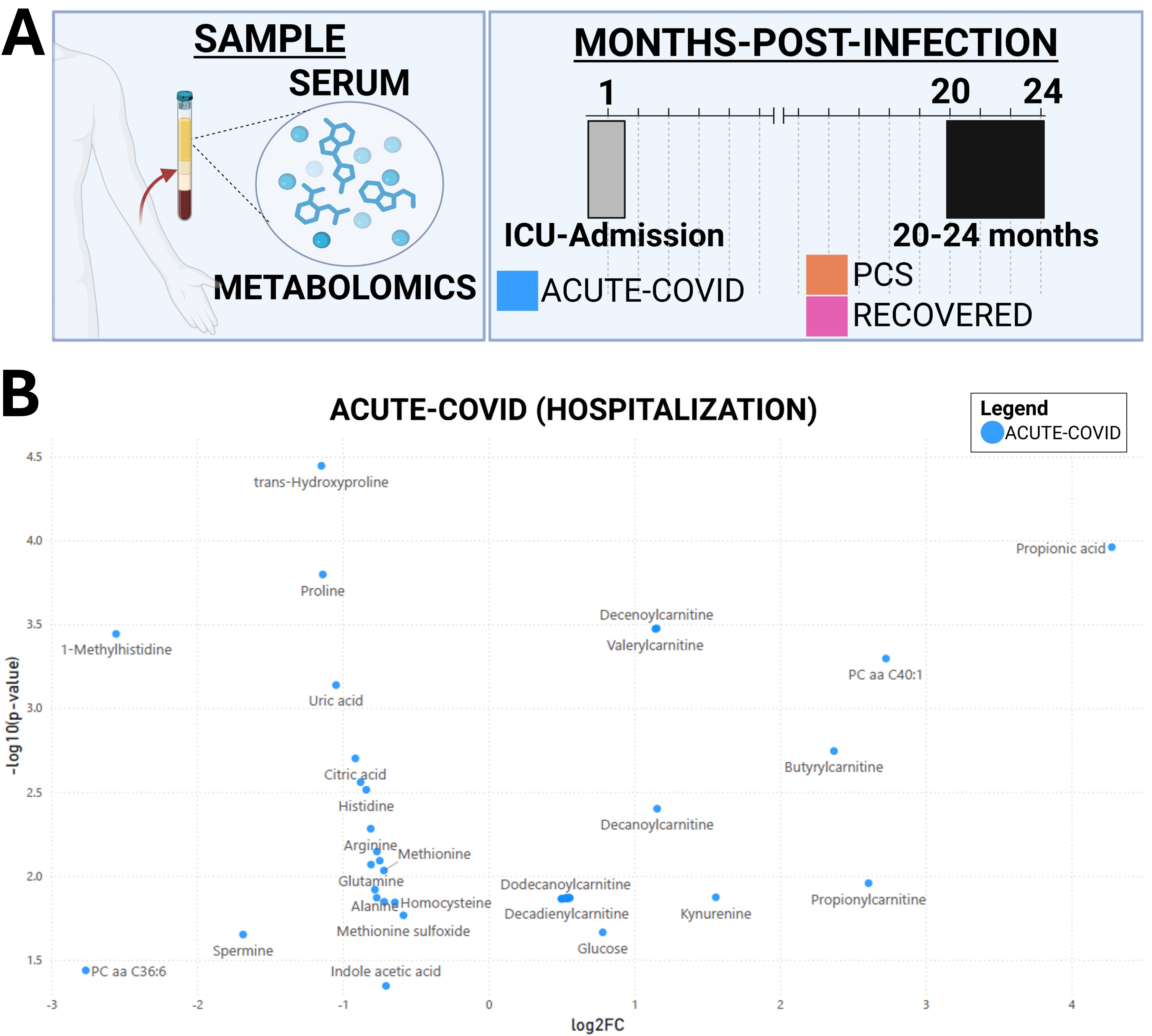
**

**Figure S5. Volcano plots of significant serum metabolic signatures in acute-COVID-19, and in PCS and recovered patients 2 years post-infection.**


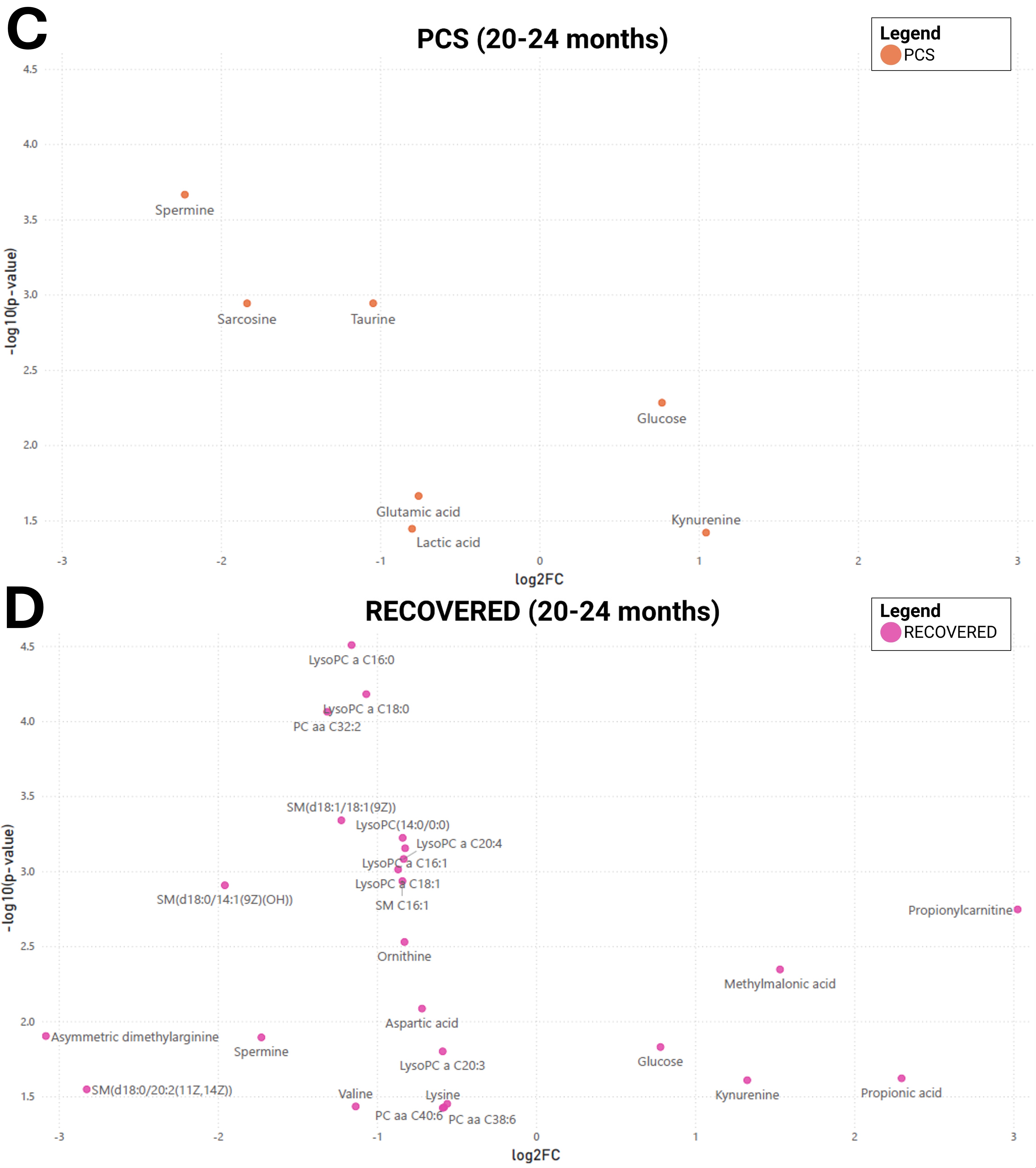


**Figure S5 (Continued). Volcano plots of significant serum metabolic signatures in acute COVID-19, and in PCS and recovered patients 2 years post-infection.** (A) Overview of human serum metabolomic datasets analyzed. (B–D) Volcano plots of log10(adjusted p-values) and log2 fold changes for all significant serum metabolites (p-value < 0.05) from (B) Acute-COVID-19 patients at hospital admission, (C) PCS patients, and (D) COVID-recovered individuals sampled 20–24 months post-infection, each compared to healthy controls. P-value threshold = 0.05. Sample sizes: healthy controls (n = 37), Acute-COVID (n = 14), PCS (n = 31), and Recovered (n = 18). All n values represent biologically independent human subjects.


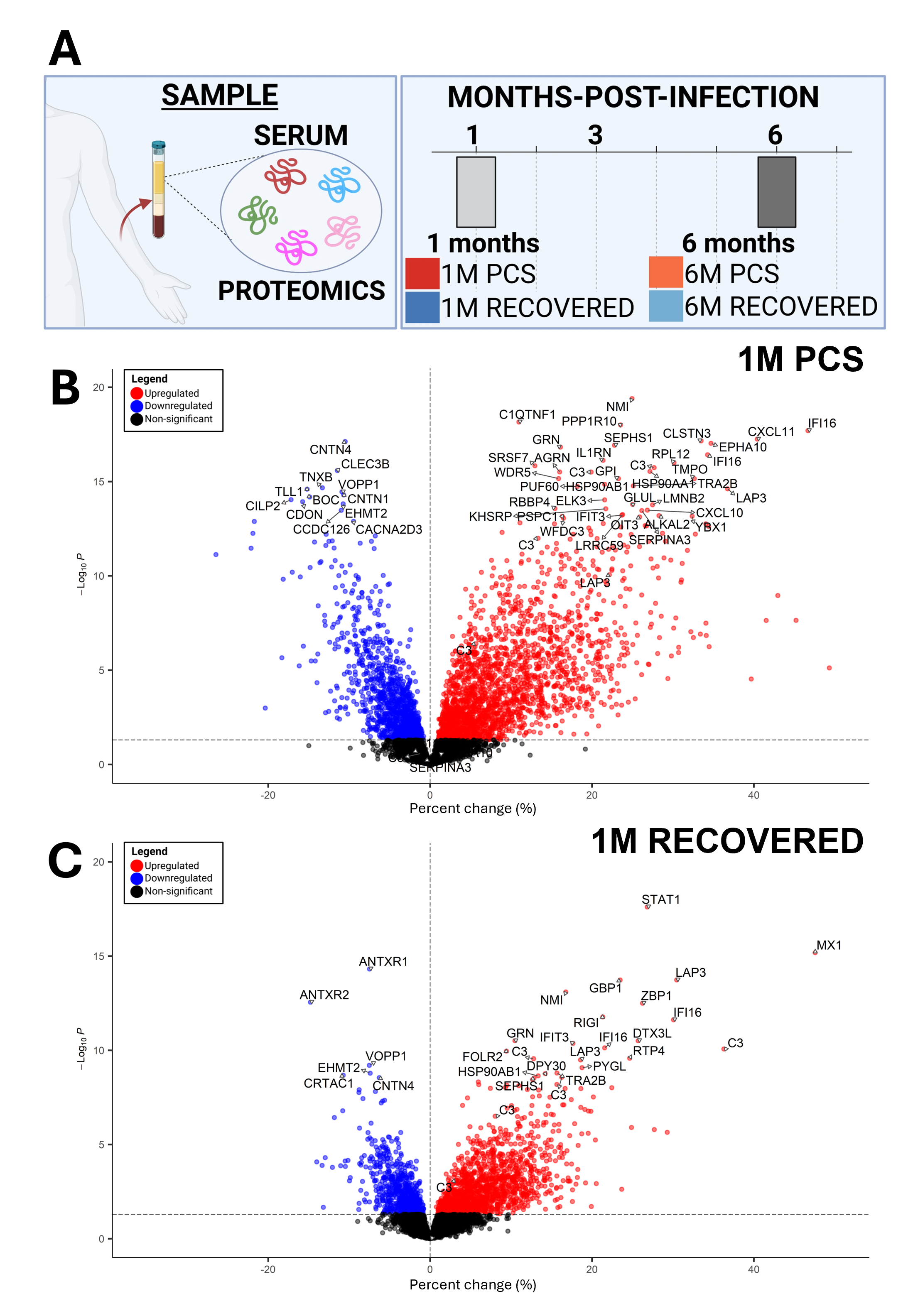


**Figure S6. Volcano plots of serum proteomic signatures in PCS and recovered patients at 1 month post-infection.** (A) Overview of human serum proteomic datasets analyzed. (B–C) Volcano plots of log10(adjusted p-values) and percent change differences for proteomic profiles comparing PCS (B) and COVID-recovered patients (C) relative to healthy controls at 1 month post infection. P-value threshold = 0.05. Sample sizes for the longitudinal serum proteomics cohort were as follows: healthy-controls (n = 39); 1M PCS (n = 40); and 1M Recovered (n = 73). Comparisons: 1M PCS vs. healthy-controls and 1M Recovered vs healthy-controls. All n values represent biologically independent human subjects.


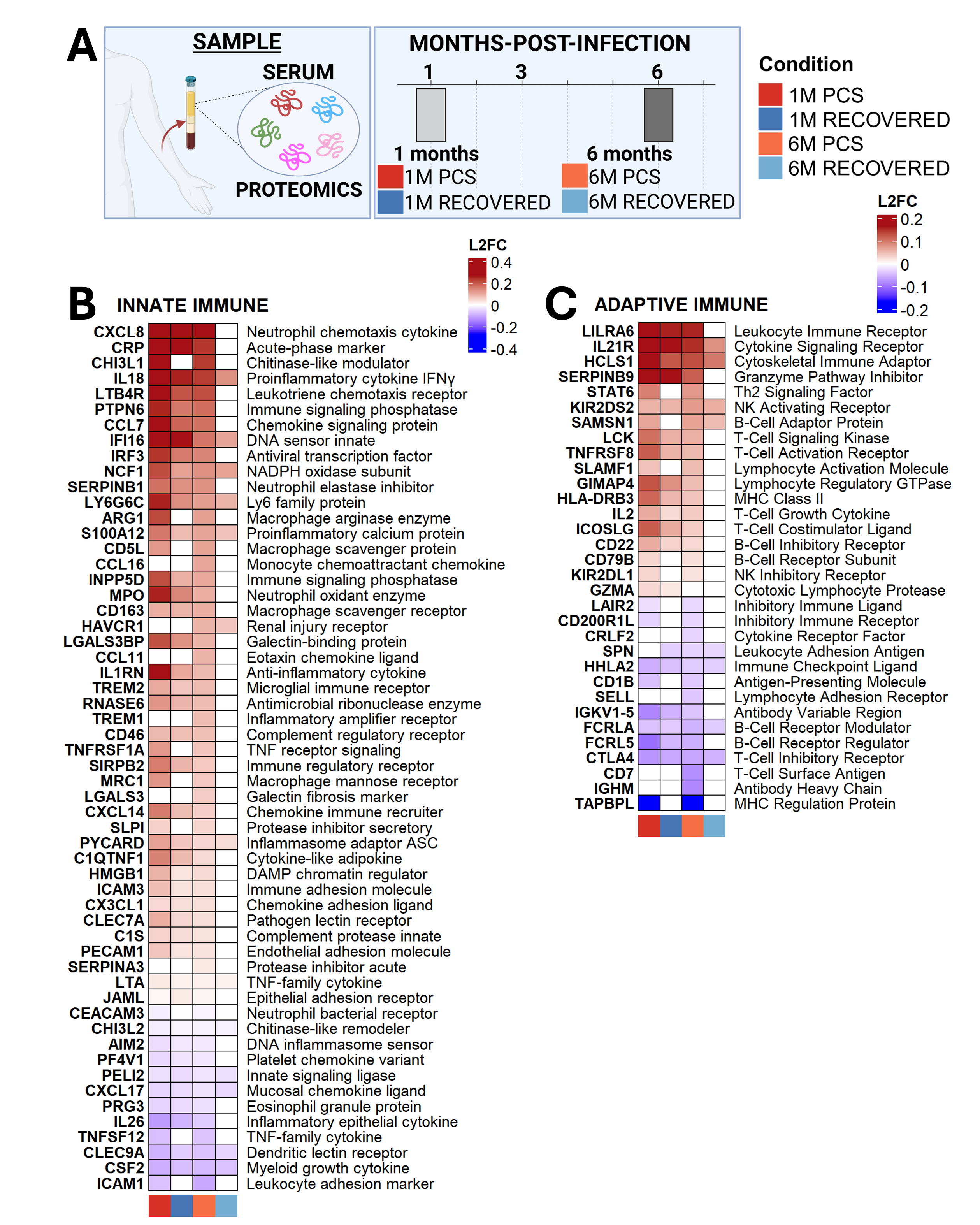


**Figure S7. Heatmaps of serum proteomic innate and adaptive immune signatures in PCS and recovered patients at 1 and 6 months post-infection.** (A) Overview of human serum proteomic datasets analyzed. (B-C) Heatmap of (C) Innate Immune and (D) Adaptive Immune proteins. P-value threshold = 0.05. Sample sizes for the longitudinal serum proteomics cohort were as follows: healthy-controls (n = 39); 1M PCS (n = 40); 6M PCS (n = 38); 1M Recovered (n = 73); 6M Recovered (n = 75). Comparisons: 1M PCS vs. healthy-controls, 6M PCS vs. healthy-controls, 1M Recovered vs healthy-controls, and 6M Recovered vs healthy-controls. All n values represent biologically independent human subjects.
